# Half-Covered ‘Glitter-Cake’ AM@SE Composite: A Novel Electrode Design for High Energy Density All-Solid-State Batteries

**DOI:** 10.1007/s40820-024-01644-6

**Published:** 2025-01-28

**Authors:** Min Ji Kim, Jin-Sung Park, Jin Woong Lee, Sung Eun Wang, Dowoong Yoon, Jong Deok Lee, Jung Hyun Kim, Taeseup Song, Ju Li, Yun Chan Kang, Dae Soo Jung

**Affiliations:** 1 Energy and Environmental Division, Korea Institute of Ceramic Engineering and Technology, Jinju, Gyeongnam, 52851 Republic of Korea; 2 Department of Materials Science and Engineering, Korea University, Anam-Dong, Seongbuk-Gu, Seoul, 136-713 Republic of Korea; 3 Department of Nuclear Science and Engineering, Massachusetts Institute of Technology, Cambridge, MA 20139 USA; 4 Department of Materials Science and Engineering, Ajou University, Suwon, 16499 Republic of Korea; 5 Department of Energy Systems Research, Ajou University, Suwon, 16499 Republic of Korea; 6 Department of Energy Engineering, Hanyang University, Seoul, 04763 Republic of Korea; 7 Department of Materials Science and Engineering, Massachusetts Institute of Technology, Cambridge, MA 02139 USA

**Keywords:** All-solid-state batteries, Cathodes, Sulfide-based solid electrolytes, Interfaces, Mechanofusion

## Abstract

All-solid-state batteries (ASSBs) are pursued due to their potential for better safety and high energy density. However, the energy density of the cathode for ASSBs does not seem to be satisfactory due to the low utilization of active materials (AMs) at high loading. With small amount of solid electrolyte (SE) powder in the cathode, poor electrochemical performance is often observed due to contact loss and non-homogeneous distribution of AMs and SEs, leading to high tortuosity and limitation of lithium and electron transport pathways. Here, we propose a novel cathode design that can achieve high volumetric energy density of 1258 Wh L^−1^ at high AM content of 85 wt% by synergizing the merits of AM@SE core–shell composite particles with conformally coated thin SE shell prepared from mechanofusion process and small SE particles. The core–shell structure with an intimate and thin SE shell guarantees high ionic conduction pathway while unharming the electronic conduction. In addition, small SE particles play the role of a filler that reduces the packing porosity in the cathode composite electrode as well as between the cathode and the SE separator layer. The systematic demonstration of the optimization process may provide understanding and guidance on the design of electrodes for ASSBs with high electrode density, capacity, and ultimately energy density.

**Supplementary Information:**

The online version contains supplementary material available at 10.1007/s40820-024-01644-6.

## Highlights

- A novel electrode design for high energy density all-solid-state batteries (ASSBs) is realized through the control of nano- and microstructures.
- An active materials@solid electrolyte composite with a half-covered “glitter-cake” morphology is adopted to optimize the transport of electrons and ions in the electrode.
- The optimized electrode design exhibited a volumetric energy density of 1258 Wh L^−1^ at an active material content of 85 wt%, exceeding the performance of the reported ASSB cells.

**Supplementary Information:**

The online version contains supplementary material available at 10.1007/s40820-024-01644-6.

## Graphical abstract

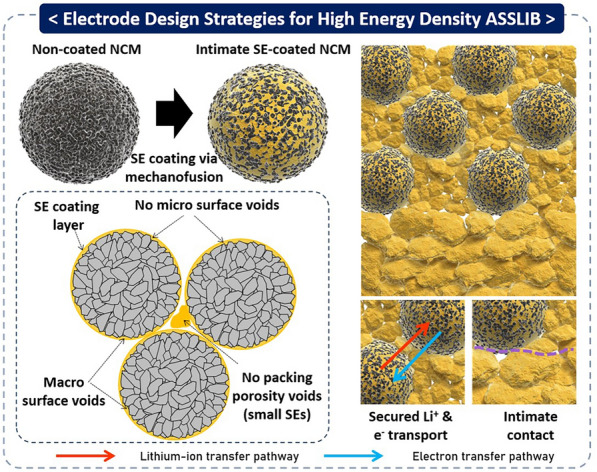

## Introduction

Lithium-ion batteries (LIBs) are currently the dominant power source for portable electronics and electric vehicles (EVs), whose importance is ever-increasing for a sustainable future [1, 2]. Unfortunately, conventional LIBs that make use of organic liquid electrolytes (LEs) and graphite anode are reaching the limitation in terms of energy density [1–6], and the flammability of organic LEs is also a concern [7, 8]. All-solid-state batteries (ASSBs) that use lithium metal anode and SEs are considered as a promising alternative for replacing the conventional LIBs and are now at the forefront of next-generation rechargeable battery research [9–13]. The ASSBs research should not only provide solution to the safety issues but also focus on surpassing the energy density of the conventional LIBs [14–17]. Among various types of SEs, sulfide-based SEs, such as Li_10_GP_2_S_12_ [18] and Li_7_P_3_S_12_ [10], have been widely applied as electrolytes for ASSBs due to their high ionic conductivity at room temperature and high ductility [19–22]. However, contrary to conventional LIBs where electrode–electrolyte contact is secured due to the fluidic nature of LEs, SEs cannot penetrate well into the electrode, resulting in insufficient contact area between cathode active materials (CAMs) and SEs [23]. The loss in physical contact hinders the lithium-ion transport due to high interfacial resistance, which may induce low initial Coulombic efficiency and poor cycle performance due to the gradual degradation of lithium-ion percolation network [24, 25]. This phenomenon becomes more obvious as the AM content increases, thereby resulting in lower lithium-ion percolation and CAM utilization, which is detrimental in terms of energy density [26–28].

To achieve ASSBs with high CAM utilization, AM loading mass, and electrode density, a favorable interface between AMs and SEs in the electrode is the key requisite [20, 24, 25, 29–32]. Much effort has been devoted to forming a highly integrable coating on the surface of AM particles as well as optimizing the size distribution of SEs. In our previous report, core–shell structured NCM@LPSCl cathode composite was successfully prepared from a mechanofusion process, where LPSCl stands for argyrodite structured Li_6_PS_5_Cl SE, and NCM stands for layer-structured Li_1-x_Ni_a_Co_b_Mn_1-a-b_O_2_ CAM [33]. Notably, each primary particle that constitutes NCM microspheres could be coated with SE, enabling the formation of a conformal coating layer. Such core–shell configuration guarantees efficient contact between AMs and SEs, which can effectively reduce void formation and agglomeration of particles, leading to ample and sufficient lithium-ion conduction pathways. In this way, ASSB system with NCM@LPSCl particles exhibited highly enhanced energy density in comparison to that with the simple mixture of NCM and SEs, where high cycle retention of 90% after 100 cycles at 0.1C was achieved with an areal capacity of 4 mAh cm^−2^. With regard to the particle size distribution, SE particles with large sizes lead to the formation of large voids and decreased contact, limiting the AM utilization yield in the electrode [34–36]. The use of smaller-sized SE particles may provide some solution since it enables the formation of lithium-ion percolation network and enhances the lithium-ion kinetics by ensuring a large contact area between AMs and SEs and homogeneity in the distribution of materials in the electrode without microvoids [37, 38].

Even though several studies provided guidance on the directions for composite electrode design, it is still far from the realization of practical ASSBs with high AM content and energy density higher than conventional LIBs [39, 40]. The battery performance dramatically degrades at AM content higher than 80 wt% due to the reduction of lithium-ion percolation [27, 38, 41]. Moreover, the electron transport pathways are limited due to the insulating characteristics of SEs, which adversely affect the rate capability of ASSBs [42–45]. Therefore, a systematic demonstration of what factors lead to the poor electrochemical performance of ASSBs and how improvements can be made should be investigated in detail.

Herein, we developed an electrode model that synergizes the effects of core–shell structured cathode composite with thin SE shell thickness and small SE particles for achieving ASSBs with high energy density and excellent electrochemical performance at a high AM loading level. Four different electrode models are proposed for step-by-step optimization of electrodes for achieving ASSBs with high energy density, where the particle distribution, porosity, ion/electron tortuosity, and electrochemical performance in each model are systematically investigated. Optimized electrode with ‘Model 4’ configuration is characterized by AM@SE core–shell structure with intimate and thin shell surrounded by small SE particles; it combines the merits of core–shell configuration with thin shell that ensures lithium-ion and electron transport and low tortuosity, and small SE particles that reduce microvoids between particles in cathode composite and between the electrode and SE separator layer. ASSBs employing such configuration exhibited a high specific capacity of 199 mAh g^−1^ with 85 wt% AM loading and volumetric energy density of 1258 Wh L^−1^, which opens up the possibility of ASSBs with high practicality.

## Experimental Section

### Materials

LiNi_0.8_Co_0.1_Mn_0.1_O_2_ (NCM811, Beijing Easpring), Li_6_PS_5_Cl (LPSCl, Creative & Innovative System), and vapor grown carbon fiber (VGCF, Sigma Aldrich) were used as AM, SE, and electronic conductive agent, respectively. Cathode electrodes were prepared as follows: Model 1 electrode was prepared from simple mixing of AM, SE, and VGCF using the agate mortar inside an argon-filled glovebox. Model 2 electrode, which comprised of core–shell structured NCM@LPSCl cathode composite, was synthesized from mechanofusion process (AMS-Mini, Hosokawa Micron Corporation) at 5000 rpm for 5 h, with NCM to LPSCl weight ratios of 75:25, 80:20, and 85:15. The operating condition of the mechanofusion process is explained in detail in our previous study [33]. To prepare Model 3 electrode, NCM@LPSCl cathode with thin SE coating layer prepared from mechanofusion process with NCM to LPSCl weight ratio of 95:5 and additional SE particles required for achieving desired AM content were mixed using the agate mortar. For the preparation of Model 4 electrode, small SE particles were obtained by planetary ball milling (Pulverisette 7 Micro Mill (Premium line), Fritsch) with 5 mm zirconia balls at 500 rpm for 3 h and subsequently mixed with the core–shell composite particles. The ionic conductivity and the particle size of the small SE particles were 2.224 mS cm^−1^ at 30 °C and 1–2 $\mu m$, respectively.

### Materials Characterization

The morphologies of the prepared cathode composite and electrodes were observed using field emission scanning electron microscopy (FE-SEM, JSM-7600F, JEOL) with energy-dispersive X-ray (EDX) spectroscopy. The 3D reconstructed and 2D sliced images were analyzed using X-ray nano-computed tomography (CT, Vtomex m 240, Baker Hughes). The analyzed area, measuring 125 × 125 × 125 $\mu m^{3} ,$ was cropped from the obtained three-dimensional composite electrode images, and image resolution of imaging battery material was adjusted to isotropic voxel pixel sizes of 400 nm. The cross section of the cathode composite was characterized by using a focused ion beam-scanning electron microscopy (FIB-SEM, Helios G4 UC, Thermo Fisher Scientific) with energy-dispersive X-ray (EDX) spectroscopy. To confirm the material phase of the prepared cathode composite, X-ray diffraction (XRD, D8 Advance, Bruker) with a Cu K $\alpha$ radiation ($\lambda$=1.542 Å) was used. The chemical nature of the prepared samples was analyzed by X-ray photoelectron spectroscopy (XPS) using a NEXSA (Thermo Fisher Scientific) with Al K $\alpha$ radiation.

### Materials Characterization

All-solid-state half-cells were fabricated with the as-prepared cathode composite particles in an argon-filled glovebox. The electrode composite powder was prepared by mixing the different as-prepared AMs with SEs and VGCF in the agate mortar, using mass fractions of 75:22.66:2.34, 80:17.66:2.34, and 85:12.66:2.34. Li metal was used as the counter electrode. First, 100 mg of LPSCl SE was placed in a ceramic mold with a diameter of 10 mm and pressed under 125 MPa. Second, the cathode composite was spread on the top of the as-prepared SE layer and pressed under 500 MPa for 30 s. The mass loading of the cathode composite was 30.6 mg cm^−2^. Finally, Li metal was attached to the other side of the SE layer and pressed under 50 MPa. Electrochemical performance was investigated using a multichannel potentiostat/galvanostat (TOSCAT3000, Toyo system, Tokyo, Japan). ASSBs were cycled at 0.05C (here, 1C = 200 mA g^−1^) between 2.50 and 4.25 V for the initial cycle and 0.2C for the following cycles. For measurement of rate capability, current density was changed step-by-step from 0.1C to 0.5C. Electrochemical properties were evaluated at 30 and 60 °C and external pressure during the operation was 15 MPa. To investigate the electrochemical performance of the NCM811 in LIBs employing liquid electrolyte, a slurry was prepared by homogeneously dispersing active material, carbon black, and PVDF in NMP at a mass fraction of 90:5:5. The half-cell was tested at 0.1C between 2.50 and 4.25 V during the initial cycle. Electrochemical impedance spectroscopy (EIS) measurements were carried out using a VSP-300 multichannel workstation over the frequency range from 7 MHz to 0.01 Hz at 20 mV after first charging and after cycles.

## Results and Discussion

### Design Principle and Structural Characterizations

**Scheme 1:**
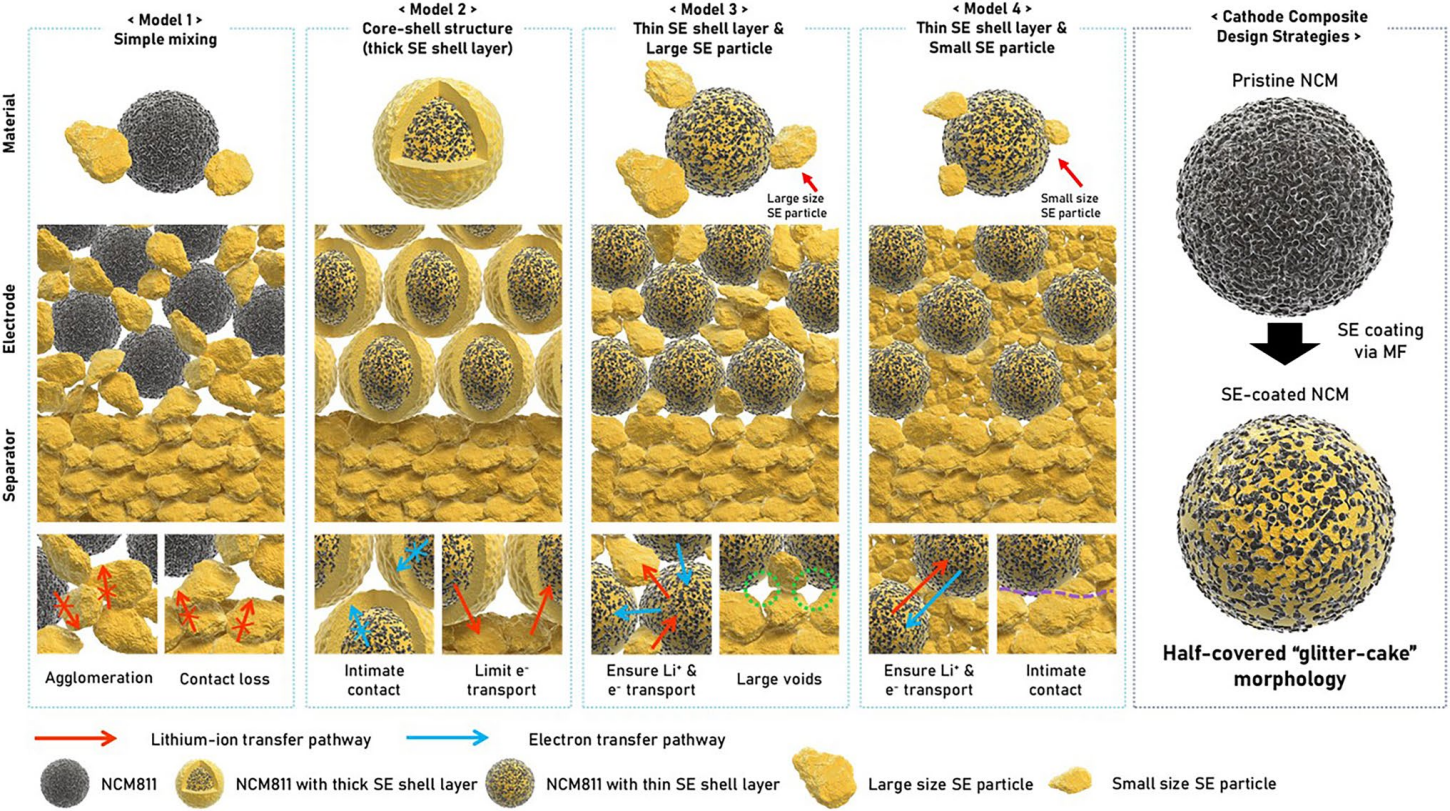
The electrode design strategies: Conventional mixing protocol (Model 1), core–shell structured cathode composite synthesized by mechanofusion process (Model 2), core–shell structured cathode composite with thin SE shell layer and large SE particle (Model 3), core–shell structured cathode composite with thin SE shell layer and small SE particle (Model 4); detailed strategy of Model 4: conformal thin SE coating layers which effectively fill the voids between AM and SE, and small SE particles which reduce the packing porosity between inter-particles

A detailed schematic illustration of four distinctive electrode models proposed for optimizing ASSB system that can achieve high energy density is provided in Scheme 1. In order to achieve optimum ASSB performance, the cathode composite needs to be a mixed ionic- and electronic conductor (MIEC). Given that LPSCl is an electronic insulator, meticulous care should be put into the microstructural engineering. ‘Model 1’ electrode is comprised of randomly mixed pristine AM and large SE particles, which can be prepared from a conventional mixing protocol. The inhomogeneous mixing results in the agglomeration of materials and contact loss between AM and SE, which leads to high interfacial resistance and an increase in ionic tortuosity. Such shortcomings can be circumvented by applying the ‘Model 2’ electrode, which utilizes core–shell structured AM@SE composite. Here, NCM@LPSCl core–shell composite microspheres were prepared from mechanofusion process, which enabled the formation of conformal SE coating on the surface. Centrifugal force is applied to press the powder samples against the chamber wall, which then experiences large shear and compression forces since they are scraped and pressed; resultantly, rigid and large particles can be easily coated with ductile and smaller counterparts. We have previously demonstrated how particle size and mechanical properties of materials play a role in forming the core–shell structured AM@SE composite during the mechanofusion process [46, 47]. Sulfide-based SE could be easily coated onto the surface of rigid AM due to their high ductility, regardless of particle size. While the difference in formability is more crucial in the fabrication of AM@SE configuration than particle size, using SE particles that are smaller in comparison to AM counterpart results in a more uniform and smoother SE coating layer. The pristine NCM microsphere consists of numerous agglomerated nano-sized primary particles characterized by rough surfaces (Fig. S1). When the mechanofusion process is employed, NCM microsphere can be coated with SE particles without damaging its original shape, even under high shear and compression forces [48]. The FE-SEM images in Fig. S2 display NCM microspheres obtained from washing the NCM@LPSCl microspheres with ethanol for the purpose of removing the SE coating layer, where the original morphology of pristine NCM microspheres was maintained. This demonstrated that rough surface of NCM particles can be completely covered with SE, indicating that the macro and micro voids between NCM particle and SE coating layer can be effectively filled, which lead to direct contact between the individual primary particles and SE. Therefore, the generation of intimate SE coating layer on the surface of NCM microspheres and their primary particles led to a homogeneous distribution of the composite in the electrode, thereby forming an ionic conductive network with low tortuosity. However, although the core–shell cathode composite ensures an intimate interfacial contact, the thick SE coating layer can restrict the electronic conduction path due to its electronically insulating characteristics. This implies that even if the ionic conduction path is secured by the core–shell structure, ASSBs may suffer from large electronic tortuosity in the electrode; accordingly, electron pathway should be reinforced to achieve high energy density. Mechanofusion process is capable of tailoring the thickness of the SE coating layer, which can directly affect electronic transport. ‘Model 3’ electrode is constituted of NCM@LPSCl core–shell composite with thin SE shell and large SE particles (which are applied in ‘Model 1’ electrode) to compensate for the decreased amount of LPSCl material due to the thin coating for achieving the desired AM content. The thin coating formed from using smaller amount of LPSCl materials during mechanofusion process resulted in the shell thickness of 184 nm, which does not completely cover up the roughness of the NCM secondary particle. Therefore, small portion of NCM primary particles is exposed at the surface, which enables direct NCM-NCM contact or NCM-VGCF-NCM contact. Resultantly, this electrode design can ensure the low tortuosity of both lithium-ions and electrons. However, due to the large size of the SE particles, microvoids are formed between the particles in the cathode and cathode/SE separator layer. Therefore, volumetric density is decreased due to the reduced electrode density and increased tortuosity, which adversely affect the ASSB performance. The introduction of small SE particles not only decreases the porosity in the cathode and between the cathode and SE separator layer, but also enhances the volumetric energy density. ‘Model 4’ electrode, which is regarded as the optimized electrode configuration, is comprised of core–shell structured cathode composite with thin shell and small SE particles. This model synergizes the merits of thin-shelled core–shell cathode composite, which can ensure both high ionic and electronic conduction paths with low tortuosity and small SE particles that act to reduce the microvoids in the cathode as well as between electrode and SE separator layer. From a series of systematic experiments, it was demonstrated that ASSBs with ‘Model 4’ electrode configuration can achieve both high electrode density and volumetric energy density. Reducing the thickness of LPSCl did matter in securing both the ionic and electronic conductivities, and how the filler LPSCl particles and VGCF are dispersed also affected the electrochemical performance and energy density. Such electrode design can therefore be regarded as half-covered “glitter-cake” packaging.

**Fig. 1:**
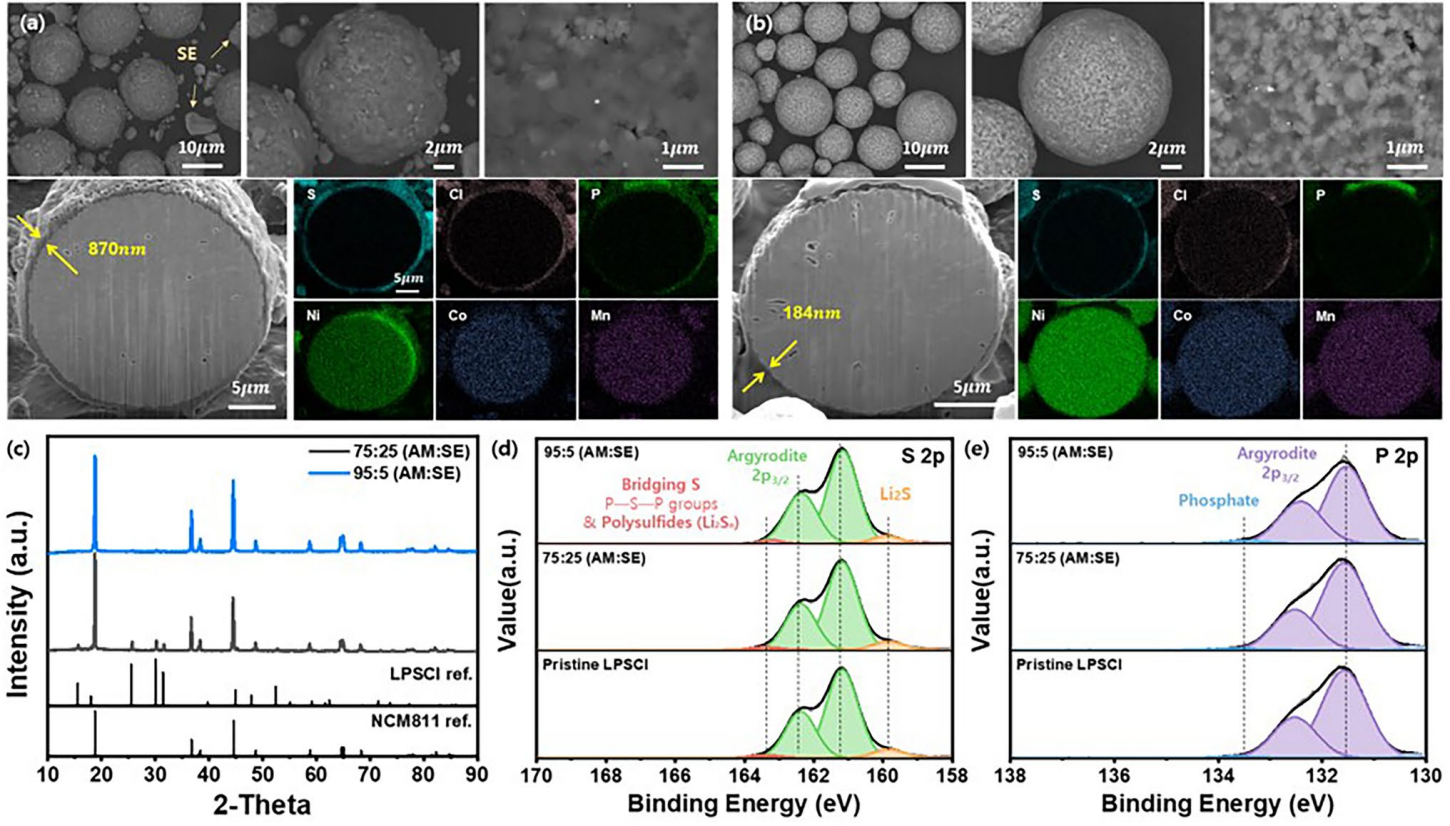
FE-SEM images with compo mode and FIB-SEM cross-sectional images of core–shell structured cathode composite: **a** 75:25 (AM:SE), **b** 95:5 (AM:SE), **c** XRD patterns of core–shell structured cathode composite, and **d** S 2*p* and **e** P 2*p* XPS spectra of pristine LPSCl and NCM@LPSCl cathode composites consisting of NCM811 and LPSCl weight ratios of 75:25 and 95:5

NCM811@LPSCl cathode composite microspheres with tailored shell thickness used for testing the electrode models were prepared from the mechanofusion process, whose morphology is shown in Fig. 1. The FE-SEM images of pristine NCM811 microspheres and LPSCl particles used in the composite electrode are shown in Figs. S1 and S3. NCM811 microspheres are characterized by spherical morphologies and highly exposed surfaces of primary particles, whereas LPSCl particles exhibit random morphology with rough surfaces. The weight ratio of NCM811 to LPSCl used for preparing core–shell composite with relatively thicker shell was 75 to 25; after the mechanofusion process, NCM particles were conformally coated with LPSCl layer, where primary particles embedded in the thick SE layer were observable. In addition, tiny SE particles that did not participate in the formation of SE shell due to the excess amount of SE used during the mechanofusion process could also be observed. The uniformity and the thickness of the shell comprising the core–shell structure were investigated with focused ion beam (FIB) SEM and elemental dot mapping. FIB-SEM images revealed that the average thickness of the SE coating layer was 870 nm, and the elemental mapping images corresponding to S, Cl, and P elements bore a resemblance to a total eclipse of the sun, meaning that dots corresponding to S, Cl, and P were only observed in the shell part. Ni, Co, Mn elements were distributed within the NCM811 microspheres, implying the successful formation of NCM811@LPSCl composite microspheres with core–shell configuration. To prepare core–shell microspheres with thinner shell thickness, the weight ratio of NCM811 and LPSCl powder used during the mechanofusion process was fixed to 95 to 5. As can be seen in Fig. S4, spherical microspheres with core–shell configuration were observed, and the tiny LPSCl particles were not detected since all LPSCl powder participated in the formation of thin shell. Even though small amount of LPSCl powder was used, the rough surface of NCM811 was covered with thin SE layer. FIB-SEM image revealed that SE layer with an average thickness of 184 nm was formed at the surface, which was further demonstrated by the elemental mapping images. SEM images and elemental mapping images of numerous particles, single particle, and magnified surface corresponding to NCM811@LPSCl composite with weight ratio of 95:5 and 75:25 were further investigated (Figs. S4 and S5, respectively). It was revealed that both microspheres exhibit core–shell configuration, where the rough surface of NCM811 was conformally coated with SE layer. However, there was an obvious difference in the thickness of the LPSCl shell that covered the primary particles with the change in AM:SE ratio. The shell thickness of NCM@LPSCl composite was calculated using the following Eq. (1), considering the weight ratio, theoretical density, and mean radius of the NCM and LPSCl. Here, the mean size of the pristine NCM particles was obtained from the particle size analysis (Fig. S6).

1 $$ \frac{{\frac{4}{3}\pi \left( {r + x} \right)^{3} - { }\frac{4}{3}\pi \left( {r - x} \right)^{3} }}{{\frac{4}{3}\pi r^{3} }} = { }\frac{{{\text{Weight}}\,{\text{of }}\,{\text{LPSCl}}}}{{{\text{Weight}}\,{\text{of}}\,{\text{NCM}}811}}{ }\frac{{\rho_{{{\text{NCM}}811}} }}{{\rho_{{{\text{LPSCl}}}} }}{ } $$

where *r* is the mean radius of the NCM (r = 3.75 $\mu m$), *x* is the shell thickness divided by 2, and *ρ* is the theoretical density (${\rho }_{NCM811}$=4.8 g cm^−3^, ${\rho }_{LPSCl}$=1.64 g cm^−3^). Thin SE layer cannot entirely cover the rough surface of NCM primary particles. Therefore, we assumed a half-covered “glitter-cake” model and used half of the thickness value for our calculations. The thickness calculated by substituting each weight and theoretical density in the equation is 880 and 181 nm for 75:25 and 95:5 NCM:LPSCl weight ratio, respectively. The calculated values coincided with the shell thickness empirically obtained from the FIB-SEM analysis. The XRD pattern of the core–shell structured cathode composite with 75:25 AM:SE ratio exhibited clear peaks relevant to NCM811 and LPSCl phases. In the case of the composite with 95:5 AM:SE ratio, minute peaks corresponding to LPSCl phase were observed due to the small amount of LPSCl used for the preparation. XPS spectra from the NCM@LPSCl composites shows only peaks related to LPSCl, such as S, P, and Cl photoemission [49], demonstrating that NCM811 particles are well covered by LPSCl (Fig. S7). The oxidation stability of LPSCl after the mechanofusion process was confirmed by S 2*p* and P 2*p* spectra as shown in Fig. 1. The intensities of all peaks are nearly identical, demonstrating that there are no chemical changes of sulfur and phosphorous in LPSCl during the mechanofusion process.

**Fig. 2:**
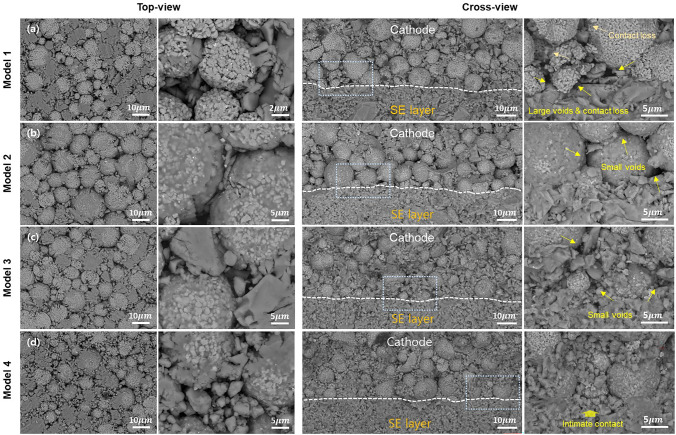
FE-SEM top-view images and cross-sectional images of the positive electrode without carbon additive: **a** Model 1 (conventional mixing), **b** Model 2 (core–shell structured cathode composite),** c** Model 3 (core–shell structured cathode composite with thin shell layer and large SE particles), and **d** Model 4 (core–shell structured cathode composite with thin shell layer and small SE particles) with 75 wt% AM loading

To elucidate the origin of the factors that affect the electrochemical properties of the composite electrode and demonstrate the ASSB system with high electrode density and volumetric energy density, Model 1–4 electrodes were prepared. Here, the small SE particles were prepared by a simple ball milling process, whose morphology is presented in Fig. S8. Top-view SEM images and the corresponding EDS mapping images of Model 1–4 with 75 wt% AM loading, and the cross-sectional SEM images of the electrodes were obtained and shown in Figs. 2 and S9. The top-view images of Model 1 electrode exhibited non-uniform distribution of AM and SE and also the contact loss between the two components. The surface contact between the cathode electrode layer and the SE separator layer was investigated with the cross-sectional SEM images, where large voids and loss of contact could also be observed. Notably, some broken pieces of NCM811 microspheres could be identified due to the brittle characteristics of AM particles; if the ductile SE particles form a uniform distribution inside the electrode or are coated on the surface, such cracks wouldn’t be observable. Top view of Model 2 electrode revealed that SE layer is conformally coated on the NCM811 microspheres with non-aggregation characteristics, and the intimate contact between each particle could be secured. However, as also shown in the cross-section SEM image, small voids between each particle were present, leaving room for improvement. The top view of Model 3 electrode revealed core–shell structured cathode composite with thinner shell in comparison to Model 2 and SE particles evenly distributed between AM@SE particles. From the cross-section view, the contact between the cathode layer and the SE separator layer was improved by the addition of SE particles that filled in the space between the core–shell particles. However, small void spaces were still present in the cathode layer due to the large size of SE particles, which prevented the formation of a dense layer. The top view and cross-sectional SEM images of Model 4 electrode revealed that the interconnected and intimate ionic and electronic percolation network could be realized by applying the core–shell particles with thin shell and small SE fillers in between. Top-view FE-SEM images and EDS mapping images of electrodes corresponding to Models 1–4 are presented in Fig. S9 to understand the distribution of AM and SE within the cathode layer. The region corresponding to the elemental mapping of Ni, Co, and Mn corresponds to the NCM microspheres, whereas S, P, and Cl elements are relevant to argyrodite particles. Model 1 electrode shows that NCM particles and large SE particles co-exist to form the cathode electrode layer, but the elemental mapping reveals the inhomogeneous distribution of AM and SE particles. In the case of Model 2 electrode, dots corresponding to Ni, Co, and Mn elements are surrounded by S, P, and Cl elements, implying the successful formation of SE coating layer on AM microspheres. Discontinuous dots corresponding to SE material suggest the presence of void space between the AM@SE microspheres, which is in line with the FIB-SEM image in Fig. S9b. Model 3 and 4 electrodes were characterized by the AM@SE particles with thin shell, which are surrounded by large and small SE particles, respectively. The percolating network was well formed in Model 3 electrode, but small voids could be observed due to the large SEs. Top-view SEM images and the corresponding EDS mapping images of Model 1–4 with 85 wt% AM loading, and the cross-sectional SEM images of the electrodes were obtained and shown in Figs. S10–S12. The results were consistent with those of 75 wt% AM loading electrodes. The core–shell structured NCM@LPSCl secured the intimate contact between AMs and SEs, leading to a compact and well-formed ionic transfer pathway in electrode. Notably, the small SE particles in Model 4 effectively filled micro and macro voids not only in the electrode but also between the electrode and SE separator, even with the small amount of SE. To confirm the distinct differences between Model 1 and Model 4, we investigated the X-ray nano-computed tomography (CT) of electrodes with 85 wt% AM loading, as shown in Fig. S13. The X-ray nano-CT analysis reconfirmed the SEM findings, highlighting that the core–shell structure and small SE particles in Model 4 ensured uniform component distribution, continuous ionic pathways, and small void volume fraction in electrode, even with low SE content, whereas Model 1 exhibited non-homogeneous distribution and large void volume fraction, leading to poor lithium-ion transfer pathways.

**Fig. 3:**
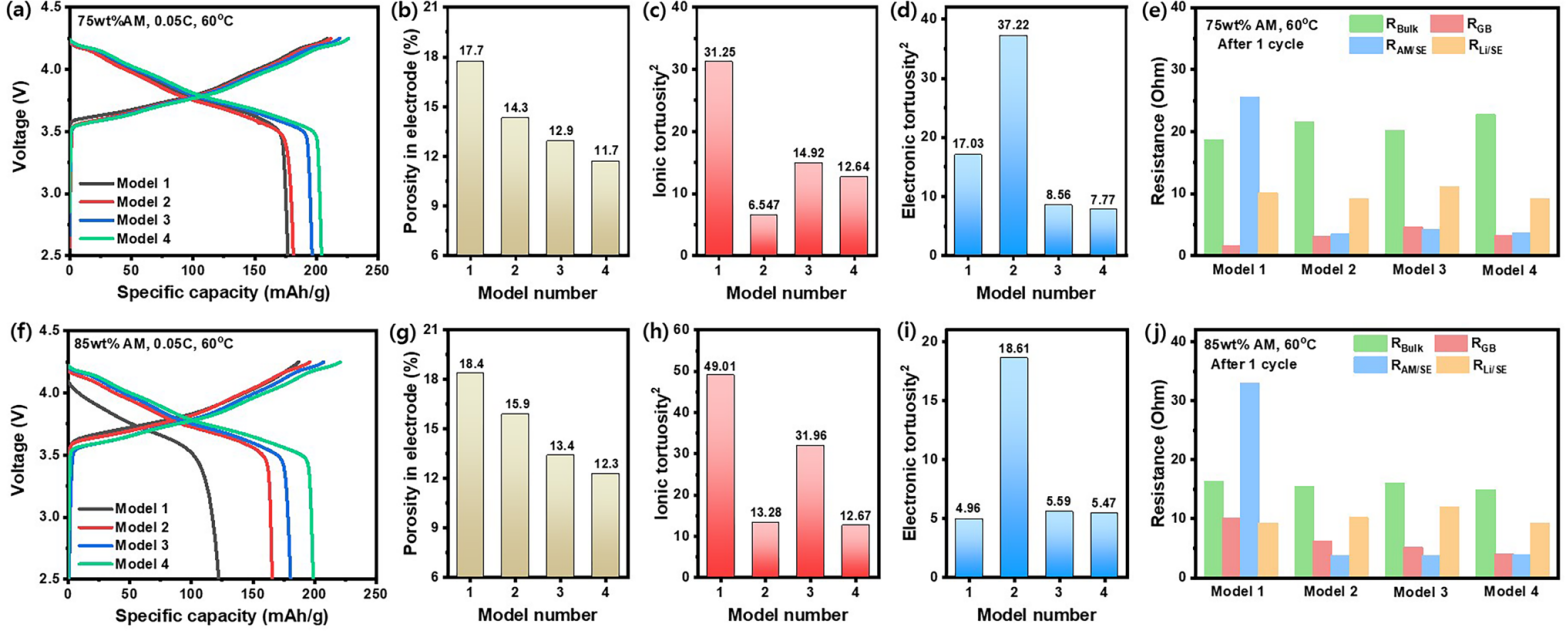
Models with 75 wt% AM loading: **a** Initial charge and discharge curves at 0.05C (1C = 200 mA g^−1^) and 60 °C for all-solid-state cathode model/Li metal half-cell. **b** Porosity in electrode. **c** Ionic tortuosity. **d** Electronic tortuosity in electrode measured by the DC polarization and **e** Resistances calculated from Nyquist plots for models after 1st cycle; Models with 85wt% AM loading: **f** Initial charge and discharge curves at 0.05C and 60 °C for all-solid-state cathode model/Li metal half-cell. **g** Porosity in electrode. **h** Ionic tortuosity. **i** Electronic tortuosity in electrode measured by the DC polarization. **j** Resistances calculated from Nyquist plots for models after 1st cycle

The LIB of half-cell employing the NCM811 exhibited a specific capacity of 204.8 mAh g^−1^ (Fig. S14), therefore, we used 200 mAh g^−1^ as theoretical capacity. The charge–discharge profiles of ASSB cells with Model 1 to 4 cathode configuration with 75 and 85 wt% AM loading (Fig. 3) were evaluated at 0.05C in the voltage range of 2.5–4.25 V (60 °C). The discharge capacities of ASSB cells showed an increasing tendency from Model 1 to Model 4 with both 75 and 85 wt% AM content, where 204.25 and 198.88 mAh g^−1^ could be delivered in Model 4 electrode, respectively. The difference in discharge capacities of each model widened as the AM content increased, owing to the smaller amount of SE component that resulted in a smaller percolating network and thus low cathode utilization. Specifically, with 85 wt% AM loading, the discharge capacities of ASSBs adopting Models from 1 to 4 were 122.19, 165.52, 180.48, and 198.88 mAh g^−1^, where a high discharge capacity could be delivered for Model 4 even at 85 wt% AM loading. The cause of the difference in discharge capacities was traced down by analyzing the porosity in electrode, and ion and electron tortuosity.

To investigate charge transfer in a cathode composite electrode, the volume fraction of each electrode material is considered. The porosity ${\Phi }_{porosity}$ of cathode electrode is defined as the volume fraction of each electrode material. The real volume fraction of each material is determined using the following Eqs. (2) and (3):

2 $$ \Phi_{{{\text{porosity}}}} = { }\frac{{V_{{{\text{meas}}}} - V_{{{\text{theo}}}} }}{{V_{{{\text{meas}}}} }}{ } $$

3 $$ { }\Phi_{i} = \left( {1 - \Phi_{{{\text{porosity}}}} } \right) \cdot V_{i} $$

where ${\Phi }_{porosity}$ is porosity in the positive electrode, ${V}_{\text{meas}}$ is calculated volume, ${V}_{\text{theo}}$ is theoretical volume, ${\Phi }_{i}$ is the real volume fractions of each electrode material and ${V}_{i}$ is the nominal volume fraction of each electrode material. The calculated porosity in the electrode is summarized in Fig. 3, where the porosity of Model 4 electrode is reduced by 6% in comparison to that of Model 1 by tailoring the configuration of constituents in the electrode. This is highly advantageous in a way that the overall energy density can be significantly enhanced. Next, the ionic and electronic tortuosity of the electrodes with Models 1–4 configuration was calculated. ${\Phi }_{i, eff}$ is the effective conductivity of a charge carrier $i$ being transported through a composite. The effective conductivity is calculated by using the geometrical thickness $L$ and area $A$ of the cylindrical composite cathode and the resistances *R*_el_ and *R*_ion_ measured by the DC polarization.

4 $$ \sigma_{{i,{\text{ eff}}}} = { }\frac{L}{{R_{i} A}}{ } $$

The tortuosity factor ${\tau }_{i}^{2}$ can be calculated using the respective effective partial conductivity ${\sigma }_{i,eff}$, the bulk conductivity ${\sigma }_{i,0}$ and the volume fraction ${\varnothing }_{i}$ of the material [50]:

5 $$ \tau_{i}^{2} = { }\frac{{\sigma_{{i,{ }0}} }}{{\sigma_{{i,{\text{ eff}}}} }}\emptyset_{i} { } $$

The ionic tortuosity of each Model is summarized in Fig. 3. The volume fraction of AM, SE, and conductive carbon additives (here, VGCF), effective conductivities, and tortuosity factors ${\tau }_{i}^{2}$ are summarized in Table S1–S4. ASSBs with 85 wt% AM content exhibited higher ionic tortuosity than 75 wt% AM content due to the lower amount of SE distributed in the cathode, while the electronic tortuosity decreased. With regard to 75 wt% AM loading, the ionic tortuosity of Model 2 shows a dramatic decrease in comparison to Model 1, which demonstrates that core–shell structured cathode composite provides a sufficient lithium-ion percolation network. However, the thick SE shell layer in Model 2 hinders electron transfer, which resulted in the highest electronic tortuosity. To overcome the insufficient electron percolation network, both Models 3 and 4 applied the AM@SE composite with thin SE shell, which resulted in low electronic tortuosity for both models since the thin shell layer thickness of 184 nm does not fully cover up the roughness of the NCM secondary particle so that the direct NCM-NCM contact or NCM-VGCF-NCM contact can be formed. Furthermore, the low ionic tortuosity of both models was ensured regardless of SE particle size due to the core–shell configuration. Here, Model 4 electrode can be regarded as a MIEC since both ionic and electronic transport are secured. For 85 wt% AM loading, in comparison to Model 1, the ionic tortuosity in Model 2 also greatly decreases due to the core–shell structure, but the electronic tortuosity greatly increases due to the thick SE shell. The ionic tortuosity of Model 3 is around 2.5 times higher than Model 4 at high AM content, which demonstrates that small SE particles can reduce the microvoids between the particles in the electrode and provide an efficient lithium-ion percolation network. Especially, both the ionic and electronic tortuosity of Model 4 with 75 and 85 wt% AM content are almost similar, thus Model 4 electrode showed a very slight decrease in terms of electrochemical performances even when AM contents increased to 85 wt%. It is notable that the design of AM@SE particles with thin SE shell can effectively reduce the electronic tortuosity and small SE particles can effectively form the ion transport pathway. The Nyquist plots of the ASSB cells with Model 1–4 electrodes were obtained after the 1st cycle at fully charged state (Fig. S15). The Nyquist plots could be deconvoluted into four types of resistance components in the system, namely, resistance of SE layer (*R*_Bulk_), grain boundary resistance of SE (*R*_GB_), interfacial resistance between AM and SE (*R*_AM/SE_), and interfacial resistance between anode and SE (*R*_Li/SE_) [51]. *R*_Bulk_ and *R*_Li/SE_ of all four Models after the 1st cycle were similar regardless of AM loading level since the same type of SE layer and Li metal were utilized (Fig. 3e, j). Not much difference in *R*_GB_ values was observed for ASSBs cells with 25 wt% SE content. However, it is notable that Model 1 with 85 wt% AM content, which is characterized by the small amount of SE particles non-homogeneously distributed in the electrode, showed the highest *R*_GB_. Among the four models, Model 1 showed the highest *R*_AM/SE_; notably, the rise in *R*_AM/SE_ was observed when AM content increased from 75 to 85 wt%, which can be attributed to the poor physical contact between AM and SE. This phenomenon was barely observed in the other three models which adopted AM@SE core–shell configuration that secured the lithium-ion transfer pathway.

**Fig. 4:**
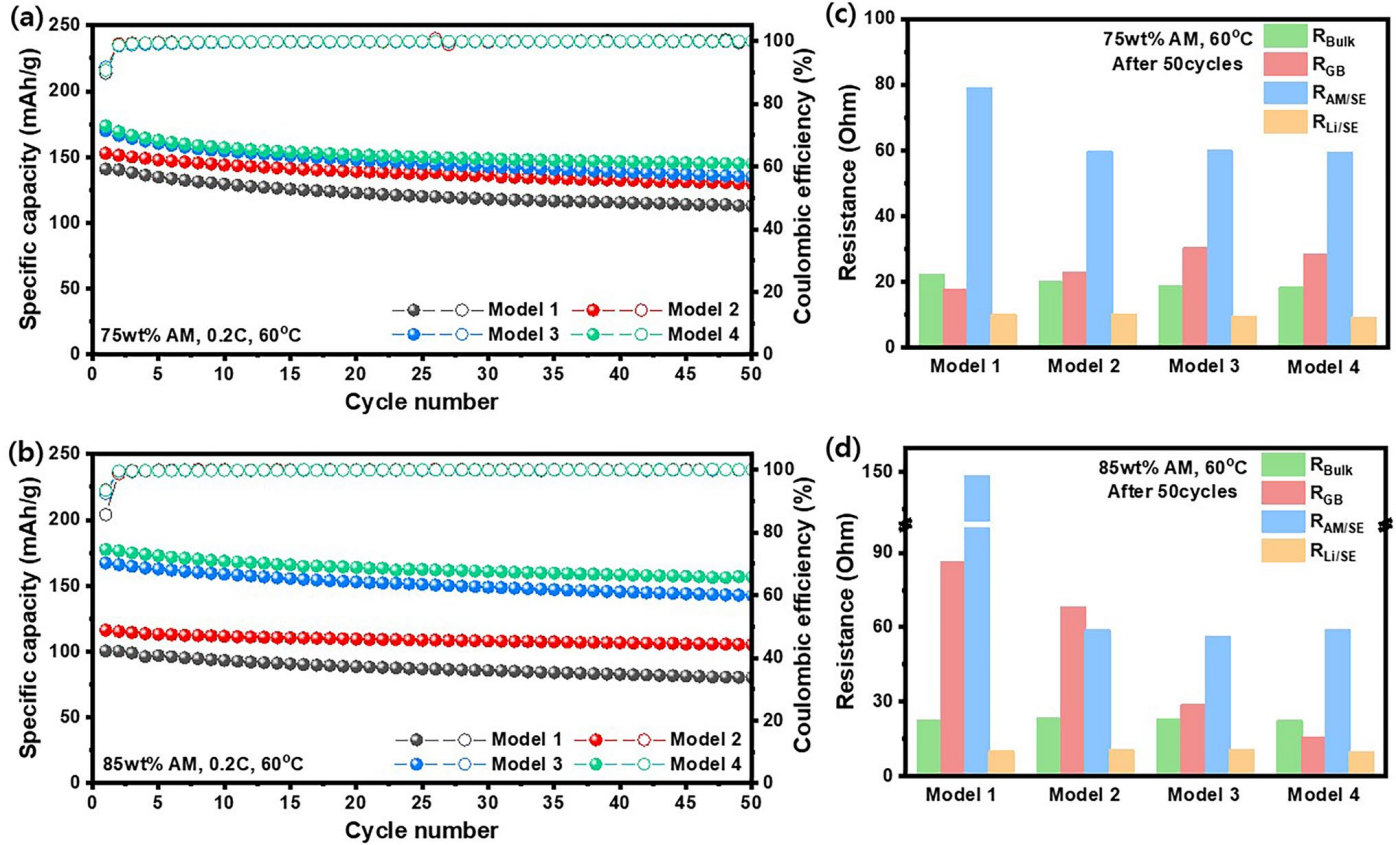
Cycle retention at 0.2C (1C = 200 mA g^−1^) and 60 °C for all-solid-state cathode model/Li metal half-cell with **a** 75 wt% AM loading and **b** 85 wt% AM loading; resistances calculated from the Nyquist plots for models after 50th cycle **c** 75 wt% AM loading and **d** 85 wt% AM loading

The cycle performance of ASSBs consisted of Model 1–4 electrode with 75 and 85 wt% AM loading were tested at 0.2C (60 °C, Fig. 4a, b). When AM loading was 75 wt%, four electrodes exhibited a similar capacity retention of ~ 76% after 50 cycles because the sufficient ion transfer pathway was formed due to a large amount of SE in electrode. However, the capacity of Model 1 with 85 wt% AM content significantly decreased at 0.2C, indicating that agglomeration and contact loss between AM and SE lead to high ionic tortuosity in the electrode. Discharge capacity of Model 2 at 0.2C for the 1st cycle was significantly decreased (116.4 mAh g^−1^) upon increasing the AM loading from 75 to 85 wt%, suggesting that electronic tortuosity plays a crucial role in improving capacity at high C-rate. Strauss et al. suggested that electrochemical performance, especially in terms of capacities, is highly affected by electronic conductivity [43]. The discharge capacity dropped dramatically with low electronic conductivity, indicating that reducing electronic tortuosity in the electrode is also essential for enhancing electrochemical performance. Both Model 3 and Model 4 exhibited high discharge capacities at 0.2C, which can be attributed to the secured electronic conductive pathway in the electrode due to the half-covered ‘glitter-cake’ AM@SE morphology with thin SE shell. Model 4 showed a higher capacity retention in comparison to Model 3 after 50 cycles, which indicates that optimization of the SE particle size for filling the porosity is also important for achieving stable cycle performance. The Nyquist plots of the ASSB cells with Model 1–4 electrodes were also obtained for the 50th cycle at a fully discharged state (Fig. S15). *R*_Bulk_ and *R*_Li/SE_ of all four Models were almost identical regardless of AM loading level (Fig. 4c, d). Model 1 with 75 wt% AM loading exhibited the smallest *R*_GB_ among the four Models, likely because the high SE content caused significant agglomeration, which may have reduced the grain boundary resistance due to the formation of continuous SE pathways. On the other hand, upon increasing the AM loading to 85 wt%, Model 1 electrode exhibited the highest *R*_GB_ value. It can be estimated that SE agglomeration also occurred in case of 85 wt% AM loading, but small amount of SE in the electrode led to a large amount of pores inside the electrode, resulting in discontinuous ion transport pathways. In Model 4 with 85 wt% AM loading, the core–shell structured cathode composite with a thin SE shell thickness and small SE particles prevented physical contact loss and maintained the integrity of electrode structure after cycling. These synergistic advantages effectively reduced overall resistivity. *R*_AM/SE_ was the highest for Model 1, where the value was even higher when AM content increased from 75 to 85 wt%. This is mainly attributed to the physical contact loss between AM and SE, suggesting that the isolation of AM particles during cycling can contribute to the capacity degradation in ASSBs. On the other hand, core–shell structured composite provides intimate contact between AM and SE, suggesting that the lithium-ion transfer pathway is secured during cycling.

The rate performance of the cells employing Model 1–4 at various C-rates were also investigated at 60 °C, as shown in Fig. S16. Model 4 constantly exhibited the highest specific capacity and rate capability with both 75 and 85 wt% AM across all C-rates, followed by Models 3, 2, and 1. This implies that both high ionic and electronic percolation network should be secured for achieving high rate capability. There was not too much difference in the rate capability between Model 1–4 with 75 wt% AM loading, which can be attributed to the sufficient ion and electron transfer pathways due to large amount of SE in the electrode. However, rate capability between Models 1–4 showed large discrepancies with 85 wt% AM loading, with capacity retention differences widening as the C-rate increased. Model 1 exhibited the lowest rate capabilities, suggesting that simple mixing does not establish a secure ion transfer pathway or sufficient contact area between the AM and SE, particularly with a limited SE amount in the electrode (Fig. 3h, j). Model 2 showed an improved rate capability over Model 1 by forming a core–shell structured AM@SE, which leads to low ionic tortuosity. Model 3 further enhanced rate capability by reducing the SE shell thickness, which could ensure low electronic tortuosity. Model 4 exhibited the highest rate capability among all Models, despite the low ionic conductivity of small SE (Figs. S17 and S18). This suggests that the synergy between half-covered ‘glitter-cake’ AM@SE composite microspheres with thin SE shell and smaller SE particles can guarantee both sufficient ionic and electronic transfer pathways in the electrode.

As proven empirically, the ionic conductivity of three types of LPSCl significantly decreases when the temperature drops from 60 to 30 °C (Figs. S17 and S18). When the electrochemical performance of ASSBs was tested at 30 °C, the cells exhibited lower discharge capacities due to a decrease in lithium-ion mobilities of argyrodite (Fig. S19). Notably, for ASSB cell with Model 2 electrode with 85 wt% AM loading, the discharge capacities at 30 and 60 °C did not show a significant difference, unlike the other three Models. Quemin et al. reported that SE degradation occurs at the interface between SE and carbon additives, with the reaction intensifying at higher temperatures [52]. Model 2 electrode is characterized by a core–shell structured AM@SE, where all SE particles were used to form a thick SE shell layer. Therefore, most of the SE is in direct contact with carbon additive, leading to substantial degradation of SE. In particular, at 60 °C (Fig. 4b), SE degradation was more pronounced compared to 30 °C (Fig. S19d), leading to lower discharge capacity at higher temperature. In particular, Model 4 with 75 and 85 wt% AM exhibited superior long-term cycling stability (Fig. S20). These are attributed to low ionic and electronic tortuosity arising from the intimate contact between AM and SE by conformal thin SE coating and low porosity caused by small SE particles.

The charge–discharge profiles of ASSBs with 80% AM loading were also analyzed at 30 °C, where the same trend of capacity increase from Model 1 to Model 4 could be confirmed (Fig. S21). All models with 80 wt% AM loading exhibit no significant difference in initial electrochemical properties compared to 75 wt% AM loading since the lithium-ion percolation network is well formed due to sufficient amount of SE. However, as AM loading increases to 85 wt%, there is a noticeable decline in the initial discharge capacity. Specifically, the degree of initial capacity fading is intensified from Model 4 to Model 1. It suggests that intimate contact and homogenous distribution of AM and SE particles in the electrode are important to achieve high ionic transfer pathways, especially at high AM loading. Moreover, a sufficient electronic conductive pathway and low porosity in the electrode and/or between the electrode and separator should be secured to increase the utilization yield of AM. Thus, electrode with appropriate design is crucial, particularly when using a small amount of SE in ASSBs.

To further demonstrate the effect of core–shell structured composite, Model 5 electrode was prepared by simply applying the Model 1 mixing protocol (conventional mixing protocol) using small SE particles. The charge–discharge profiles of ASSBs with Model 5 electrode that adopted 75, 80, and 85 wt% AM loading were analyzed at 30 °C (Fig. S22). The discharge capacities of ASSB cells with Model 5 electrode were larger in comparison to Model 1 at 75 and 80 wt% AM content since small SE particles could decrease the porosity in the electrode. However, the discharge capacity rapidly decreased when the AM content increased to 85 wt%.

**Fig. 5:**
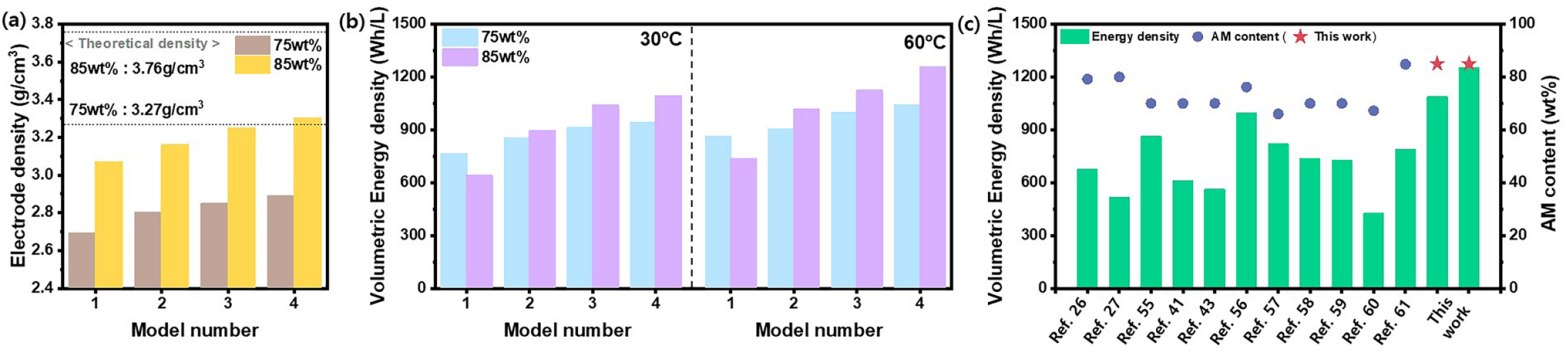
**a** Electrode density of models (NCM811 = 4.8 g cm^−3^, LPSCl = 1.64 g cm^−3^, VGCF = 2 g cm^−3^). **b** Calculation based on volumetric energy density (Wh L^−1^) of models with 75 and 85 wt% AM loading at 30 and 60 °C. Lithium metal (counter electrode) thickness of 30 $\mu m$ and separator layer thickness of 30 $\mu m$ were assumed. **c** Comparison in volumetric energy density and active material content (data of Reference from Table S6)

The electrode density and the volumetric energy densities of Models 1–4 were calculated based on the measured thickness of the electrode and the initial capacity (Fig. 5); in these calculations, it was presumed that the thickness of both the Li metal anode and separator was 30 $\mu m$ [53, 54]. The volumetric and gravimetric energy densities of Models 1–4 are presented in Supporting Information (Table S5). Model 4 exhibits the highest energy densities among all models, regardless of AM loading and temperature due to its high initial capacity and high electrode density. The details of volumetric energy density are shown in Supporting Information (Table S6) [26, 27, 41, 43, 55–61]. Irrespective of AM loading mass and the temperature, electrodes with higher Model numbers exhibited enhanced electrode density and volumetric energy density, which is attributed to the reduced porosity and increase in capacity. Notably, modification of the electrode to Model 4 enabled the achievement of a considerably higher electrode density, which accounts for 88.4% and 87.7% of the theoretical values, respectively. When the AM loading increased from 75 to 85 wt%, the volumetric energy density of Model 1 electrode without the core–shell structure sharply decreased since sufficient Li-ion percolation is not well achieved, leading to a decrease in capacity. The volumetric energy density and active material content of ASSB cells with Model 4 electrode achievable at 30 and 60 °C are compared with those reported in the previous literature (Table S6). ASSB cells with electrode comprised of core–shell structured AM@SE composite with thin shell and small SEs exhibited volumetric energy density of 1094 and 1258 Wh L^−1^ at AM content of 85 wt%, exceeding the performance of the reported ASSB cells. It is generally known that the in solvent-free process, significantly less binder is required in comparison to the wet process. The structural integrity is uniformity secured, even with a small amount of binder content. The low binder content minimizes the impact on ionic and electronic pathways, ensuring the lithium-ion and electron transport pathway in electrode. Therefore, we consider that by applying our electrode design and the solvent-free process, practical and high energy density ASSBs can be realized.

## Conclusions

Here, a novel cathode design for achieving ASSBs with high electrode density and volumetric energy density at high AM loading is proposed. Four different electrode models were proposed for step-by-step optimization of the electrodes in ASSBs: 1) A simple mixture of AM and SEs, 2) AM@SE composite particles with thick shell, 3) AM@SE composite particles with thin shell and large SE particles, and 4) AM@SE composite particles with thin shell and small SE particles. Systematic analyses were carried out to understand the correlation between the particle distribution, porosity, and ionic/electronic tortuosity in each electrode and the electrochemical performance. The optimized electrode combined the merits of the core–shell structure with thin shell that ensures both the ionic conduction between AM and SE and electronic conduction, and the small size of the SE particles that can yield highly dense electrode, which can result in high ionic and electronic conduction pathways and low tortuosity. Such design of optimized electrode led to a high volumetric energy density of 1258 Wh L^−1^, which can provide guidance on the design of novel electrodes for ASSBs with high practicality in terms of volumetric energy density.

## Supplementary Information

Below is the link to the electronic supplementary material.

Supplementary file1 (DOCX 13132 KB)
