# Supplementary material for: Half-Covered ‘Glitter-Cake’ AM@SE Composite: A Novel Electrode Design for High Energy Density All-Solid-State Batteries
Source: Nanomicro Lett. 2025 Jan 28;17:119. doi: 10.1007/s40820-024-01644-6 (PMC11775378; doi:10.1007/s40820-024-01644-6)
Supplement: Supplementary file 1 — Supplementary file1 (DOCX 13132 KB) [file 40820_2024_1644_MOESM1_ESM.docx]

Supporting Information for

**Half-Covered ‘Glitter-Cake’ AM@SE composite: A Novel Electrode Design for High Energy Density All-Solid-State Batteries**

Min Ji Kim^1, 2, #^, Jin-Sung Park^3, 4, #^, Jin Woong Lee^1, 2^, Sung Eun Wang^1, 2^, Dowoong Yoon^1, 2^, Jong Deok Lee^1, 2^, Jung Hyun Kim^1^, Taeseup Song^5^, Ju Li^3, 6, *^, Yun Chan Kang^2, *^, Dae Soo Jung^1, *^

^1^ Energy and Environmental Division, Korea Institute of Ceramic Engineering and Technology, Jinju, Gyeongnam, 52851, Republic of Korea

^2^ Department of Materials Science and Engineering, Korea University, Anam-dong, Seongbuk-gu, Seoul, 136-713, Republic of Korea

^3^ Department of Nuclear Science and Engineering, Massachusetts Institute of Technology, Cambridge, MA 20139, USA

^4^ Department of Materials Science and Engineering, Ajou University, Suwon, 16499, Republic of Korea

^5^ Department of Energy Engineering, Hanyang University, Seoul, 04763, Republic of Korea

^6^ Department of Materials Science and Engineering, Massachusetts Institute of Technology, Cambridge, MA 02139, USA

*^#^*Min Ji Kim and Jin-Sung Park contributed equally to this work.

*Corresponding authors. E-mail: [dsjung@kicet.re.kr](mailto:dsjung@kicet.re.kr) (Dae Soo Jung); [yckang@korea.ac.kr](mailto:yckang@korea.ac.kr) (Yun Chan Kang); [liju@mit.edu](mailto:liju@mit.edu) (Ju Li)

**Supplementary Figures and Tables**


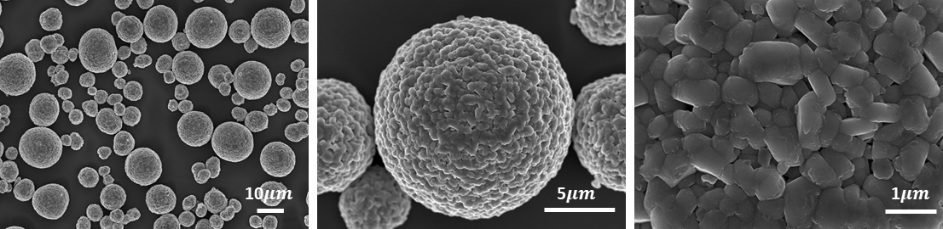


**Fig. S1** FE-SEM images of pristine NCM811


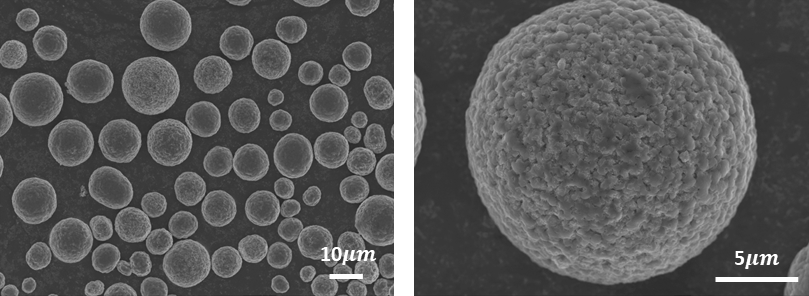


**Fig. S2** FE-SEM images of NCM811 microspheres obtained from washing the NCM@LPSCl microspheres with ethanol to remove the SE coating layer. NCM@LPSCl cathode composite consisting of NCM811 and LPSCl weight ratios of 95:5


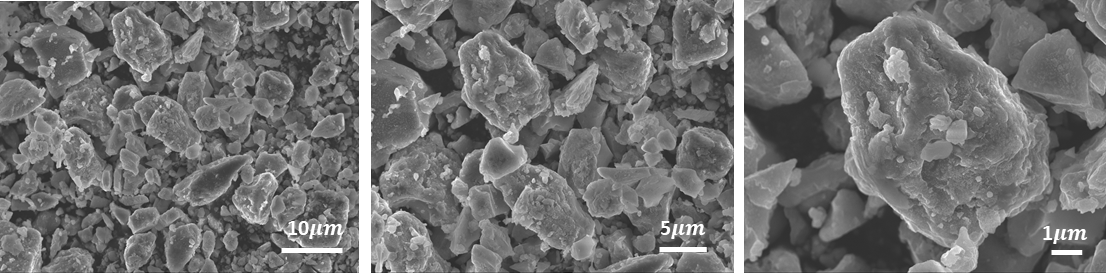


**Fig. S3** FE-SEM images of pristine LPSCl


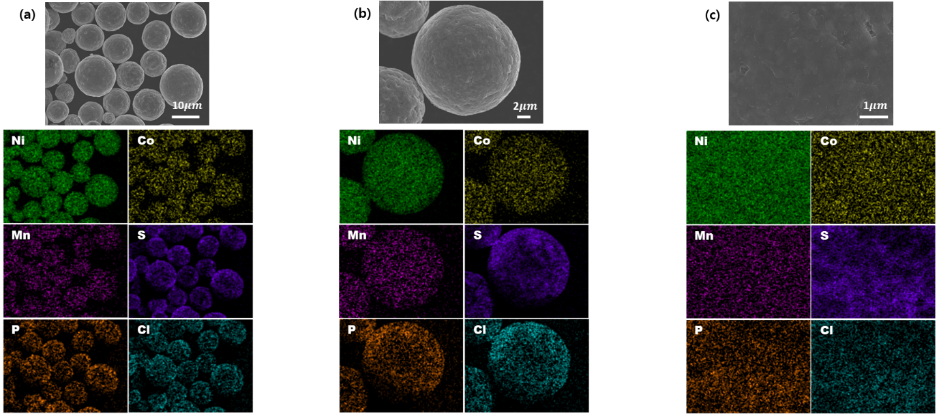


**Fig. S4** 95:5 (AM:SE) core-shell structured cathode composite FE-SEM images and EDS mapping


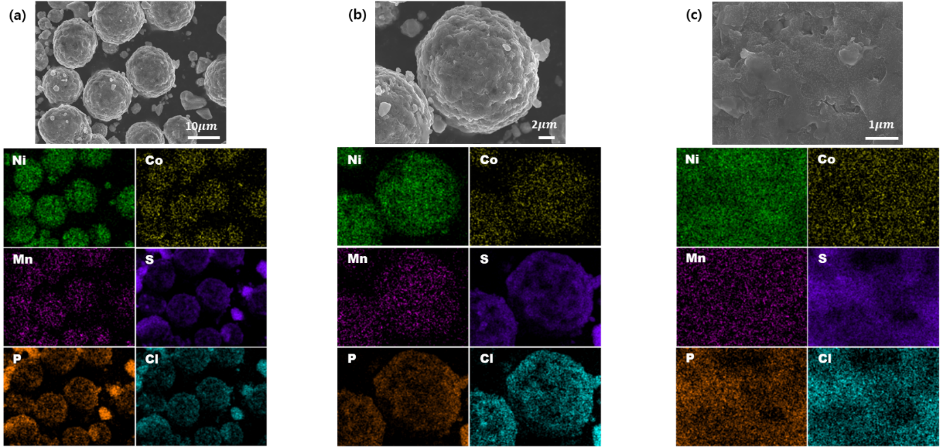


**Fig. S5** 75:25 (AM:SE) core-shell structured cathode composite FE-SEM images and EDS mapping

| **Pristine NCM811** | |
| --- | --- |
| *D*_10_ | 6.22 $\mu m$ |
| *D*_50_ | 7.37 $\mu m$ |
| *D*_100_ | 11.56$\mu m$ |
| Mean size | 7.49$\mu m$ |


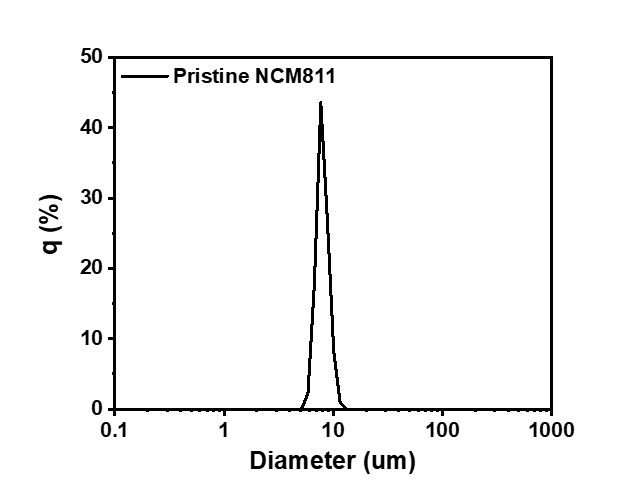


**Fig. S6** PSA analysis of pristine NCM811


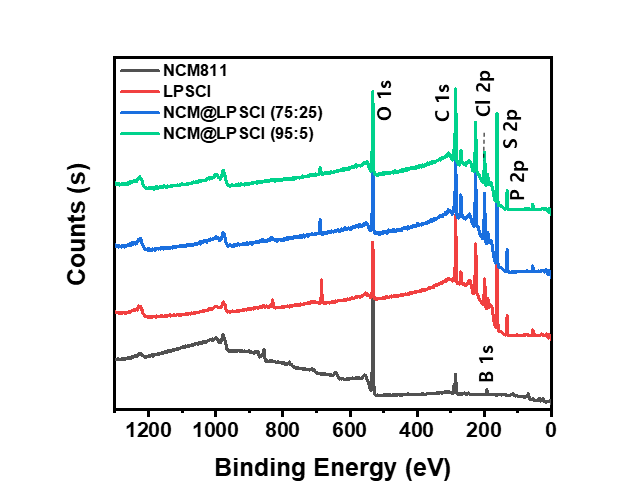


**Fig. S7** XPS spectra of pristine NCM811, pristine LPSCl and NCM@LPSCl composites. The boron-coated NCM cathode active material can suppress SE decomposition and parasitic reactions at the interface between the SE and AM, even under direct contact, thereby improving cycling stability [S1]

**Fig. S8** FE-SEM images of milled LPSCl (500rpm, 3h, 5mm ZrO_2_ ball)


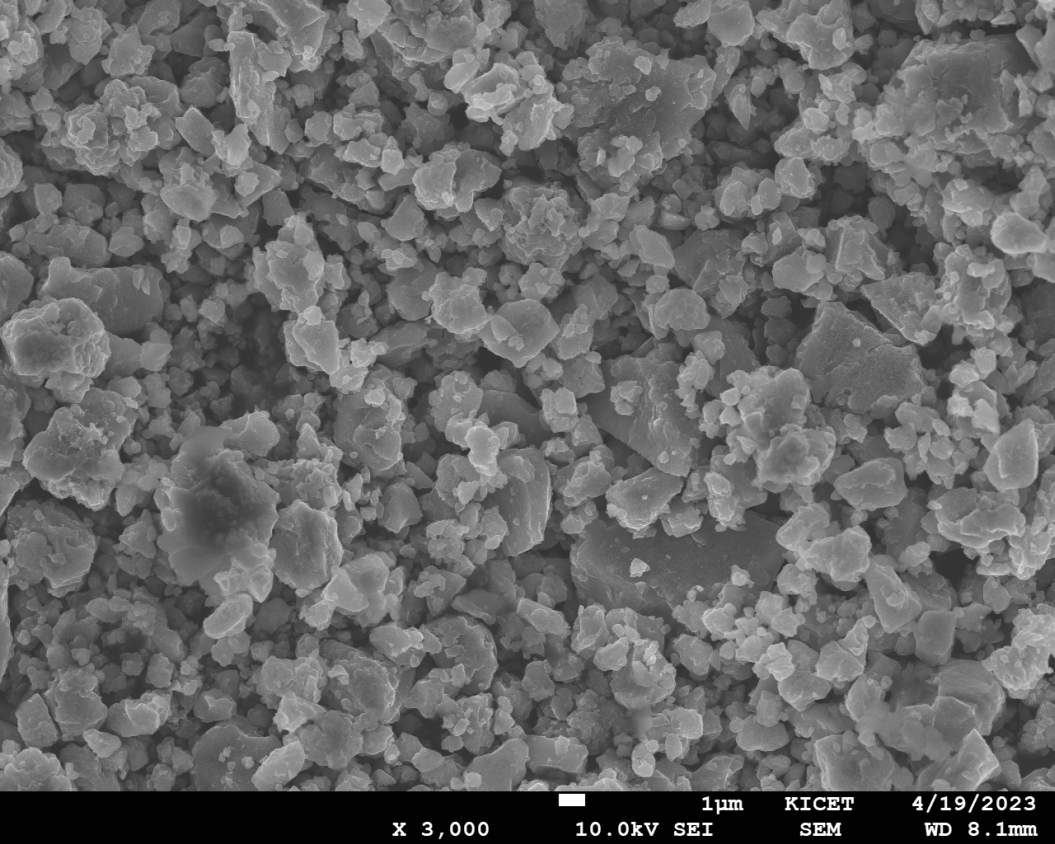

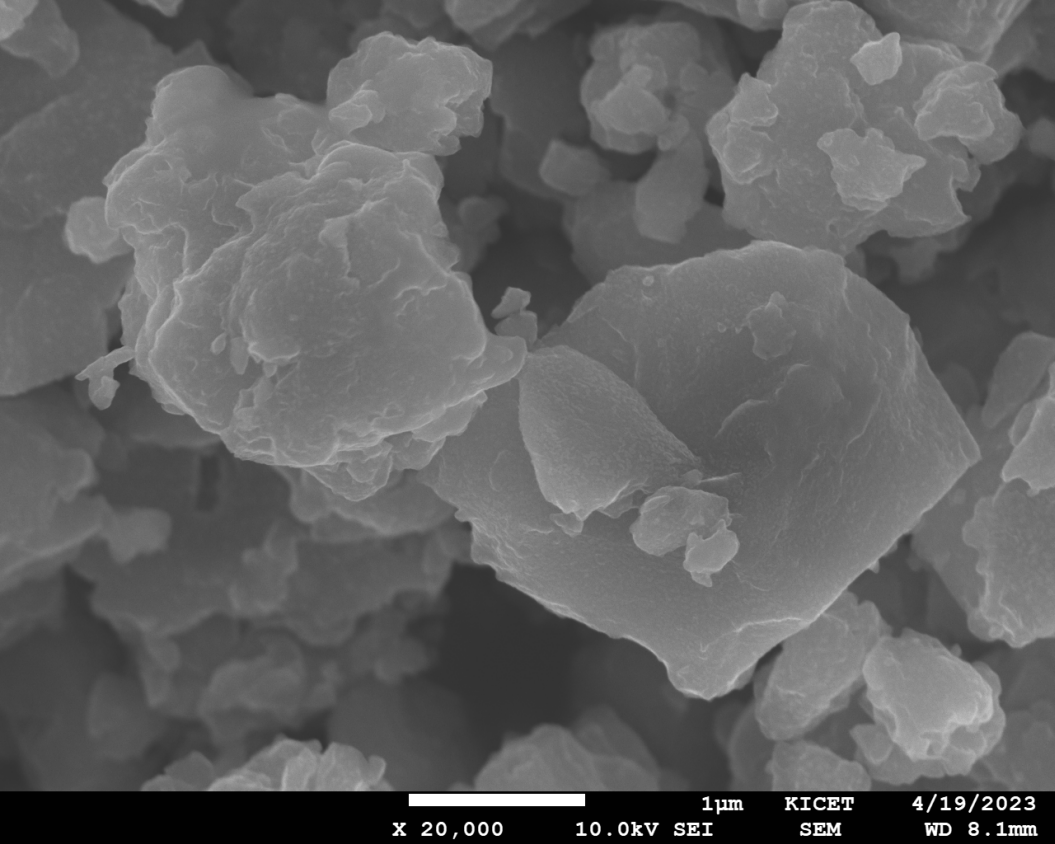


**5**$\boldsymbol{\mu m}$

**1**$\boldsymbol{\mu m}$


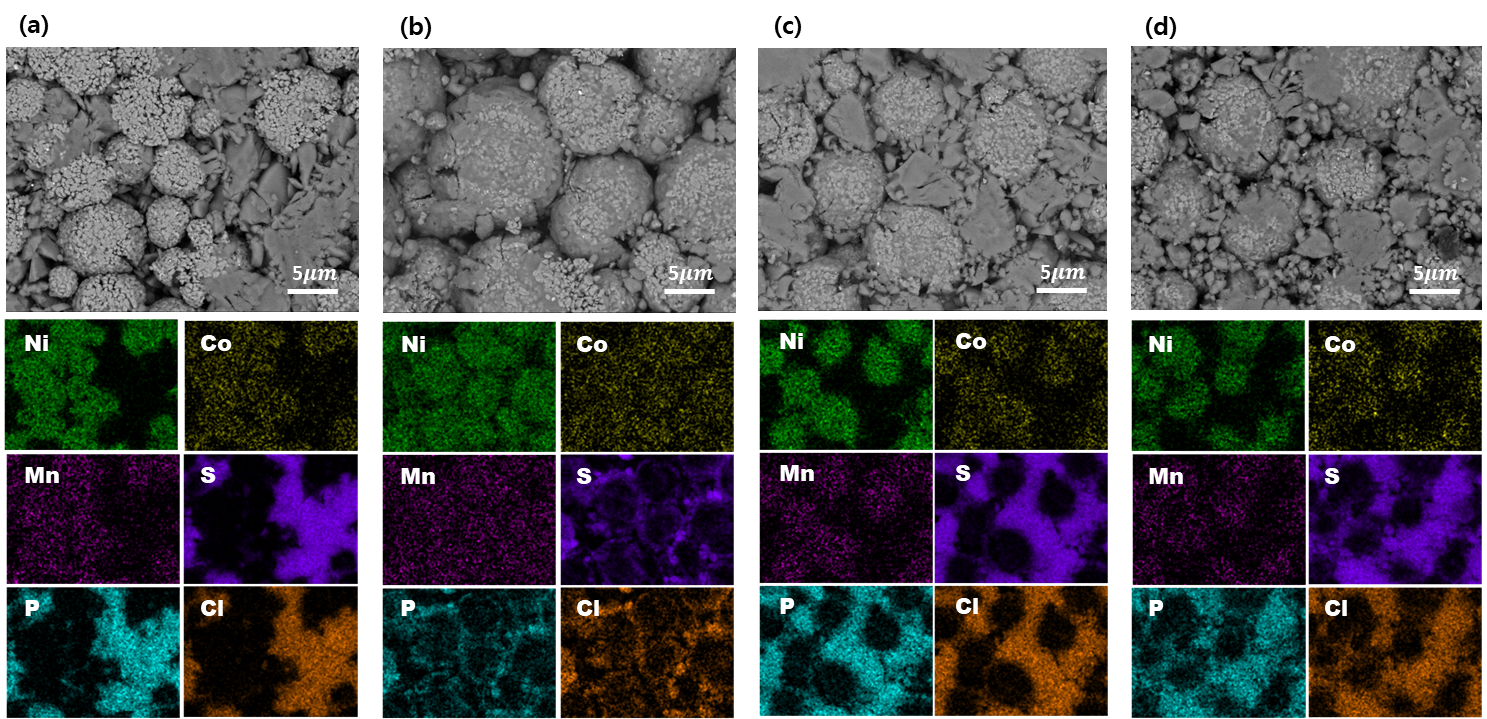


**Fig. S9** FE-SEM top-view images and EDS mapping: **a** Model (conventional mixing), **b** Model 2 (core-shell structure), **c** Model 3 (core-shell structured cathode composite with thin shell layer and large SE particles), and **d** Model 4 (core-shell structured cathode composite with thin shell layer and small SE particles) with 75 wt% AM loading


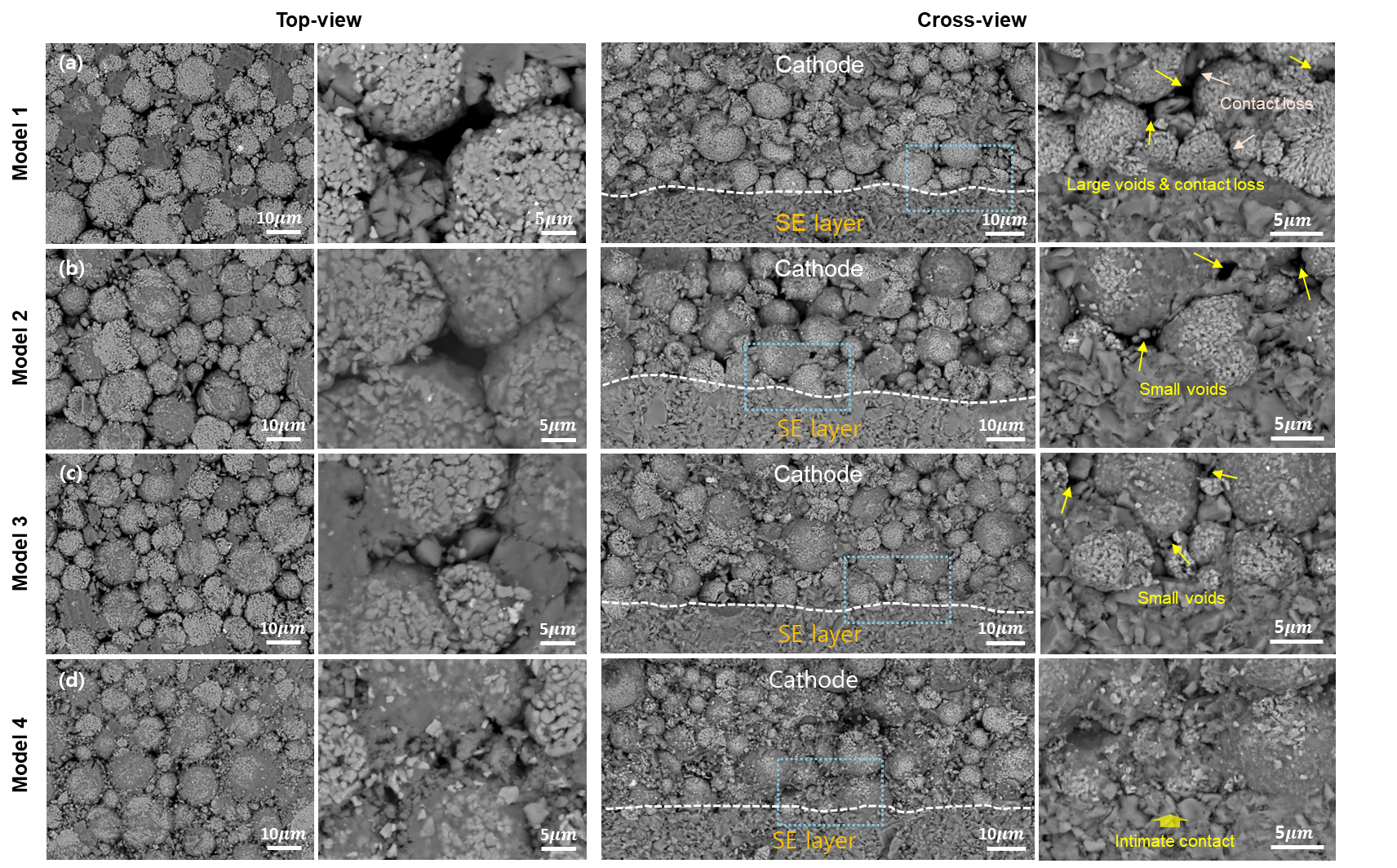


**Fig. S10** FE-SEM top-view images and cross-sectional images of electrode without carbon additive: **a** Model 1 (conventional mixing), **b** Model 2 (core-shell structured cathode composite), **c** Model 3 (thin shell coating layer and large SE particle), and **d** Model 4 (thin shell coating layer and small SE particle) with 85 wt% AM loading


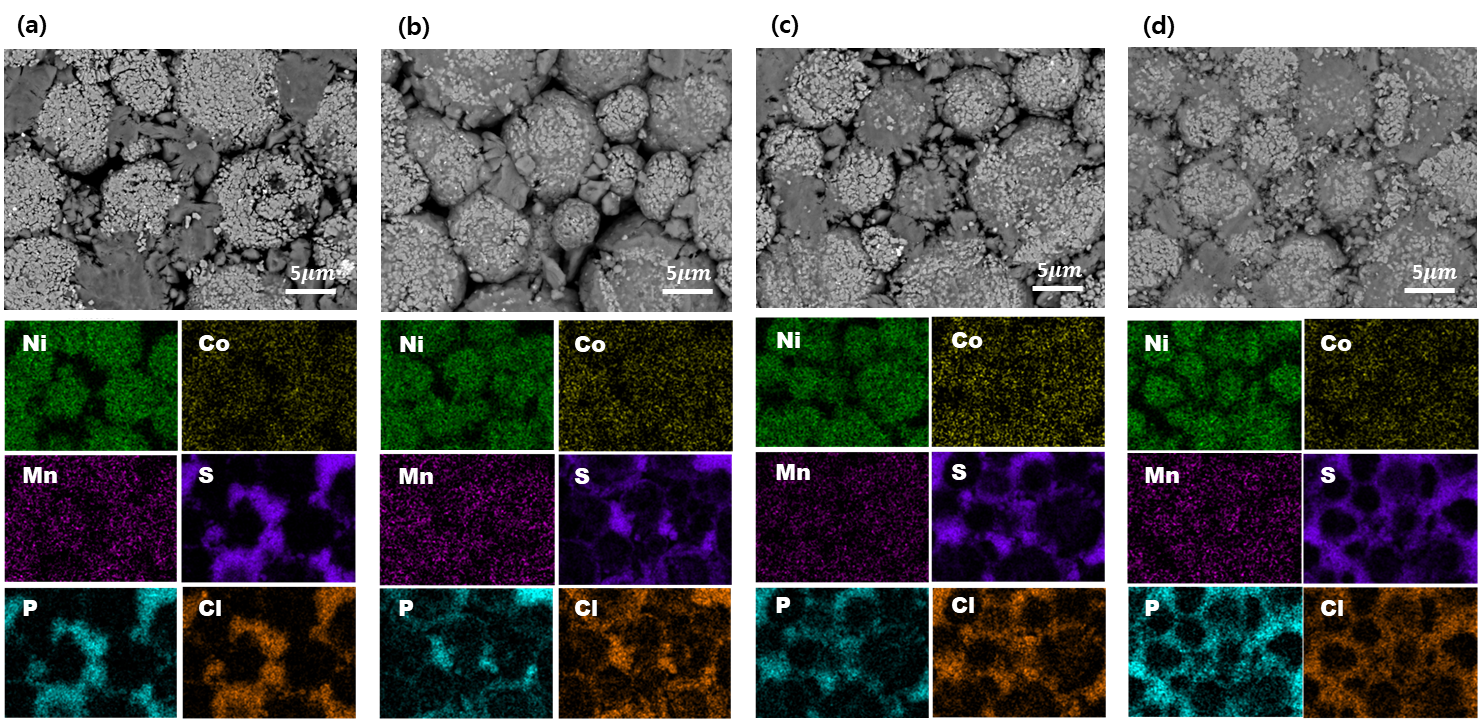


**Fig. S11** FE-SEM top-view images and EDS mapping: **a** Model 1 (conventional mixing), **b** Model 2 (core-shell structured cathode composite), **c** Model 3 (thin shell coating layer and large SE particle), and **d** Model 4 (thin shell coating layer and small SE particle) with 85 wt% AM loading


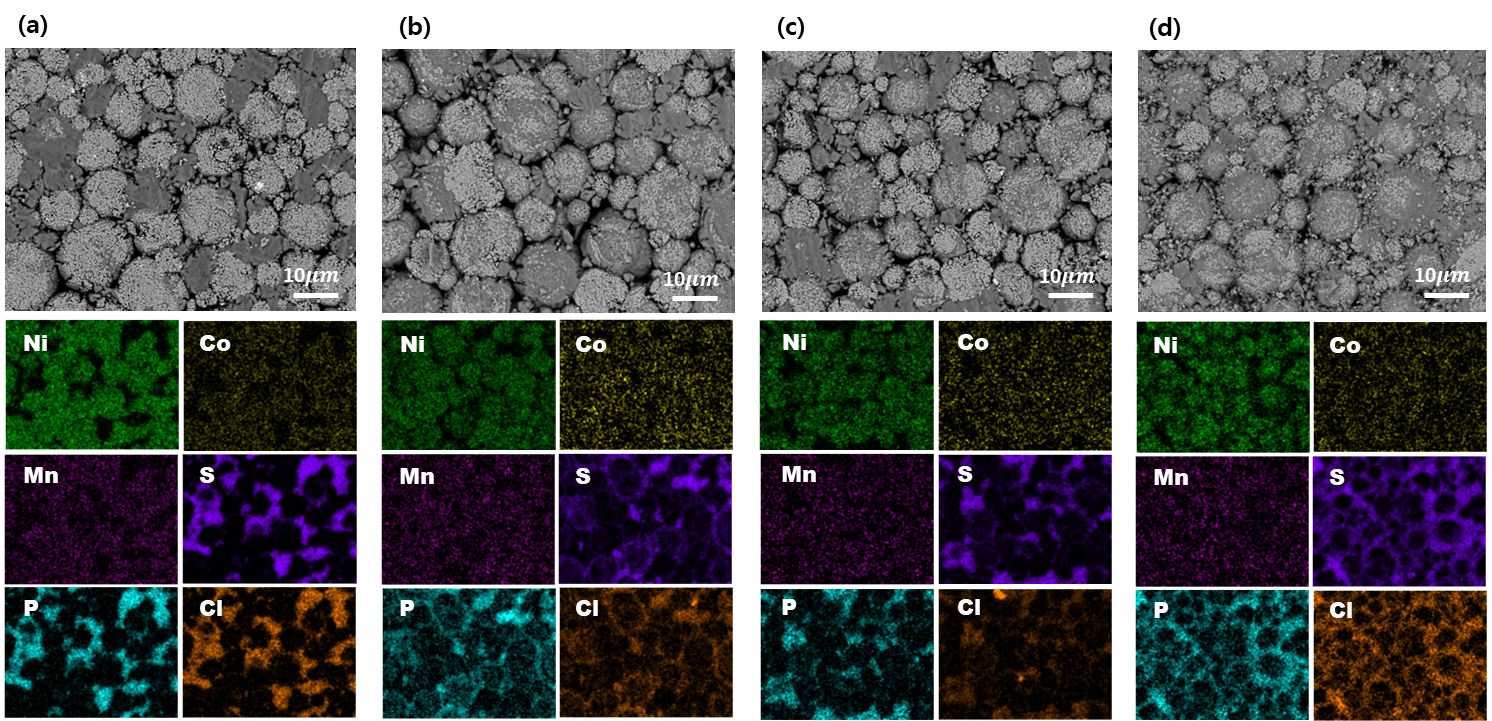


**Fig. S12** FE-SEM top-view images and EDS mapping at low magnification: **a** model 1 (simple mixing), **b** model 2 (core-shell structure), **c** model 3 (thin shell coating layer), **d** model 4 (small solid electrolyte particle) with 85wt% AM loading


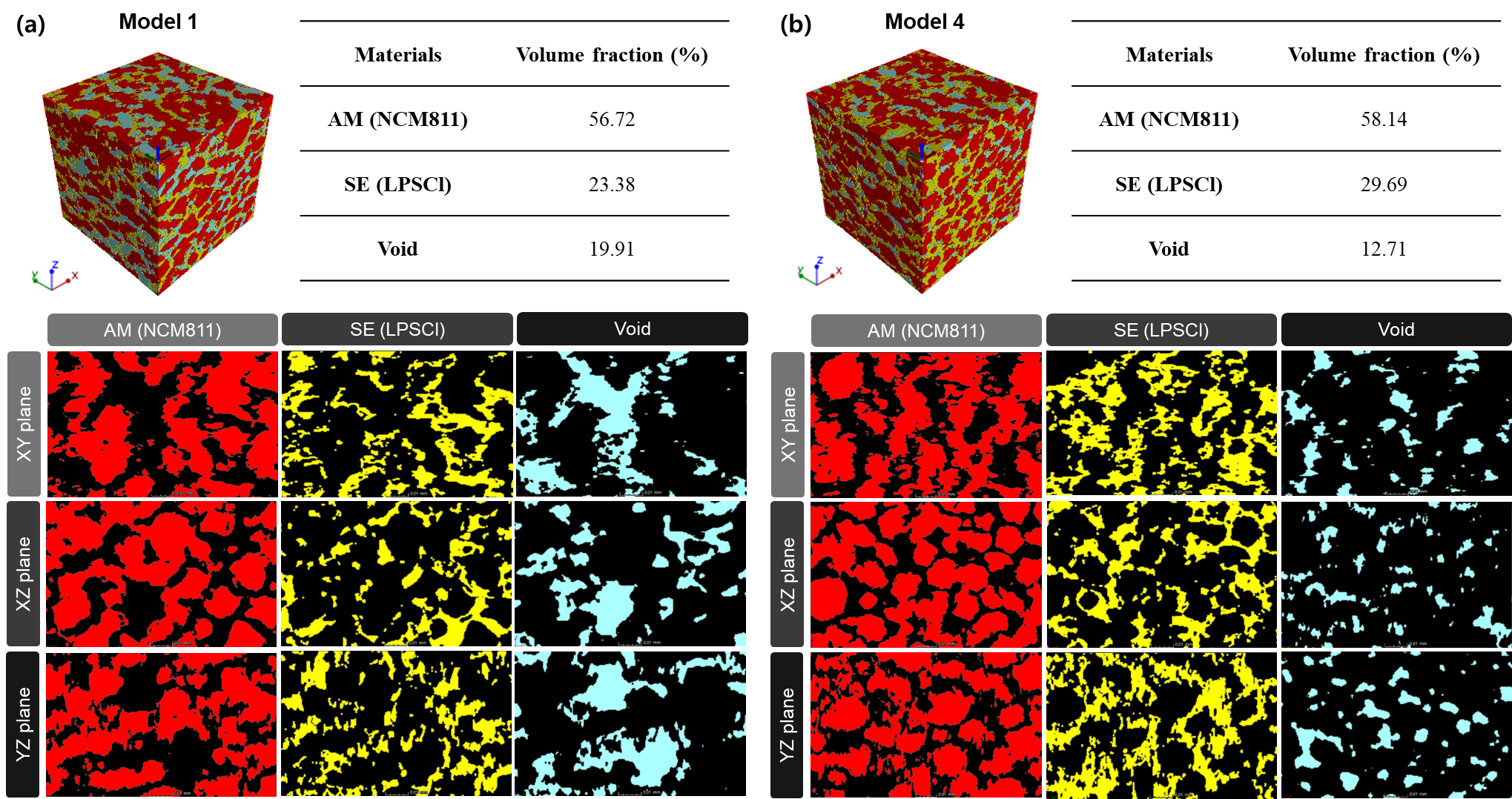


**Fig. S13** Reconstructed nano-CT images of a Model 1 and b Model 4 with 85 wt% AM loading. The reconstructed 3D volume of the electrodes (125ⅹ125ⅹ125 μm^3^) was segmented into three phases (red: AM (NCM811); yellow: SE (LPSCl), blue: void) based on gray-scale thresholding. Through the segmentation process, the volume fractions of different components in the electrodes were determined. The 2D slice images showed segmentation results of individual components (AM, SE, and void) in the x-y, x-z, and y-z planes

**Fig. S14** Charge-discharge profile for the 1^st^ cycle at 0.1C for NCM811 cathode/Li half-cell

| **AM 75wt%** | ***Φ_SE_* / %** | ***σ*_ion, eff_ / mS cm^-1^** | ***τ*_ion_^2^** |
| --- | --- | --- | --- |
| Pristine SE | 100 | 14.82 | 1.00 |
| Model 1 | 37.04 | 0.176 | 31.25 |
| MF SE | 100 | 10.53 | 1.00 |
| Model 2 | 38.66 | 0.617 | 6.547 |
| Pristine SE | 100 | 14.82 | 1.00 |
| Model 3 | 39.46 | 0.392 | 14.92 |
| Milled SE | 100 | 5.704 | 1.00 |
| Model 4 | 39.87 | 0.180 | 12.64 |

**Table S1** Volume fraction of SE determined effective conductivities σ_eff_ and tortuosity factors τ^2^ of the ionic transport pathway. Tortuosity factors have been set to unity for the pristine materials for a simplification of the analysis

**Table S2** Volume fraction of AM and CA(conductive additive) determined effective conductivities σ_eff_ and tortuosity factors τ^2^ of the electronic transport pathway. Tortuosity factors have been set to unity for the pristine materials for a simplification of the analysis

| **AM 75wt%** | ***Φ*_NCM_ / %** | ***Φ*_VGCF_ / %** | ***σ*_electron, eff_ / mS cm^-1^** | ***τ*_electron_^2^** |
| --- | --- | --- | --- | --- |
| Model 1 | 41.99 | 3.14 | 0.264 | 17.03 |
| Model 2 | 43.72 | 3.27 | 0.125 | 37.22 |
| Model 3 | 44.46 | 3.33 | 0.561 | 8.56 |
| Model 4 | 44.58 | 3.34 | 0.623 | 7.769 |
| NCM + VGCF | 97.66 | 2.34 | 10 | 1.00 |

**Table S3** Volume fraction of SE determined effective conductivities σ_eff_ and tortuosity factors τ^2^ of the ionic transport pathway. Tortuosity factors have been set to unity for the pristine materials for a simplification of the analysis

| **AM 85wt%** | ***Φ_SE_* / %** | ***σ*_ion, eff_ / mS cm^-1^** | ***τ*_ion_^2^** |
| --- | --- | --- | --- |
| Pristine SE | 100 | 14.82 | 1.00 |
| Model 1 | 23.68 | 0.088 | 49.01 |
| MF SE | 100 | 10.53 | 1.00 |
| Model 2 | 24.57 | 0.195 | 13.28 |
| Pristine SE | 100 | 14.82 | 1.00 |
| Model 3 | 25.09 | 0.116 | 31.96 |
| Milled SE | 100 | 5.704 | 1.00 |
| Model 4 | 25.64 | 0.115 | 12.67 |

**Table S4** Volume fraction of AM and CA(conductive additive) determined effective conductivities σ_eff_ and tortuosity factors τ^2^ of the electronic transport pathway. Tortuosity factors have been set to unity for the pristine materials for a simplification of the analysis

| **AM 85wt%** | ***Φ*_NCM_ / %** | ***Φ*_VGCF_ / %** | ***σ*_electron, eff_ / mS cm^-1^** | ***τ*_electron_^2^** |
| --- | --- | --- | --- | --- |
| Model 1 | 54.33 | 3.590 | 1.431 | 4.956 |
| Model 2 | 56.02 | 3.701 | 0.330 | 18.61 |
| Model 3 | 57.63 | 3.808 | 1.096 | 5.593 |
| Model 4 | 58.37 | 3.857 | 1.146 | 5.473 |
| NCM + VGCF | 97.66 | 2.34 | 10 | 1.00 |


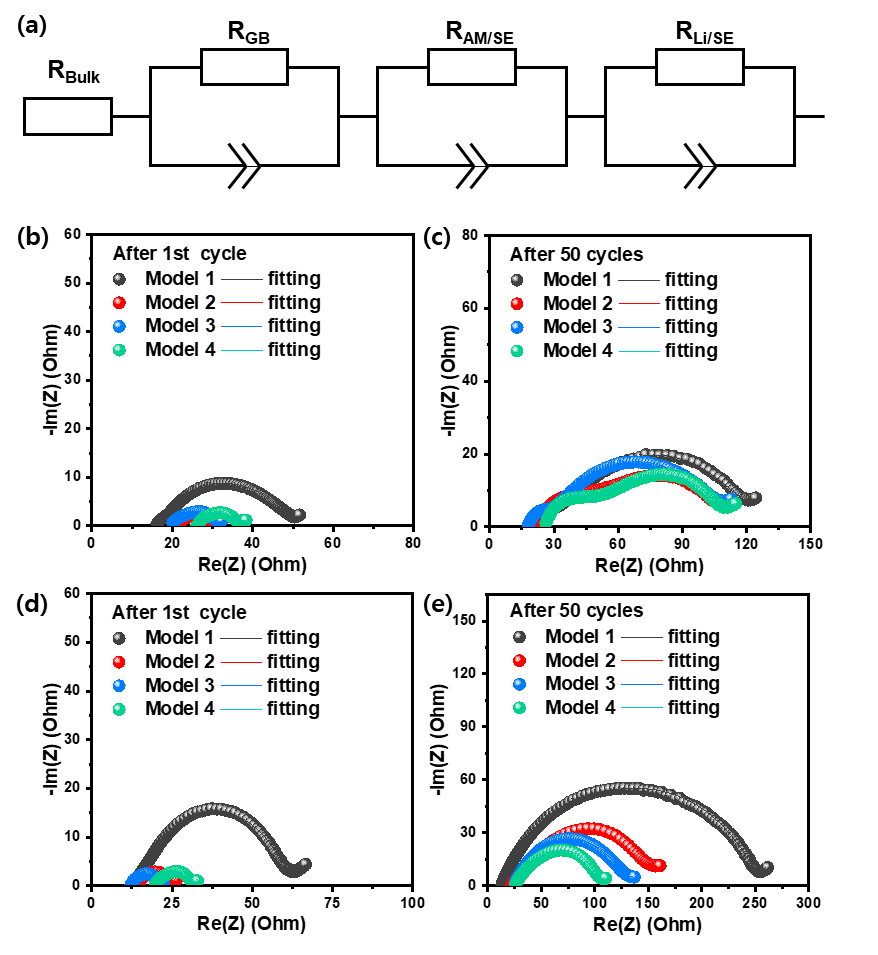


**Fig. S15** **a** Equivalent circuit used to fit the experimental data, Nyquist plots, **b** 1^st^ cycle with 75wt% AM loading, **c** 50^th^ cycle with 75wt% AM loading, **d** 1^st^ cycle with 85wt% AM loading and **e** 50^th^ cycle with 85wt% AM loading


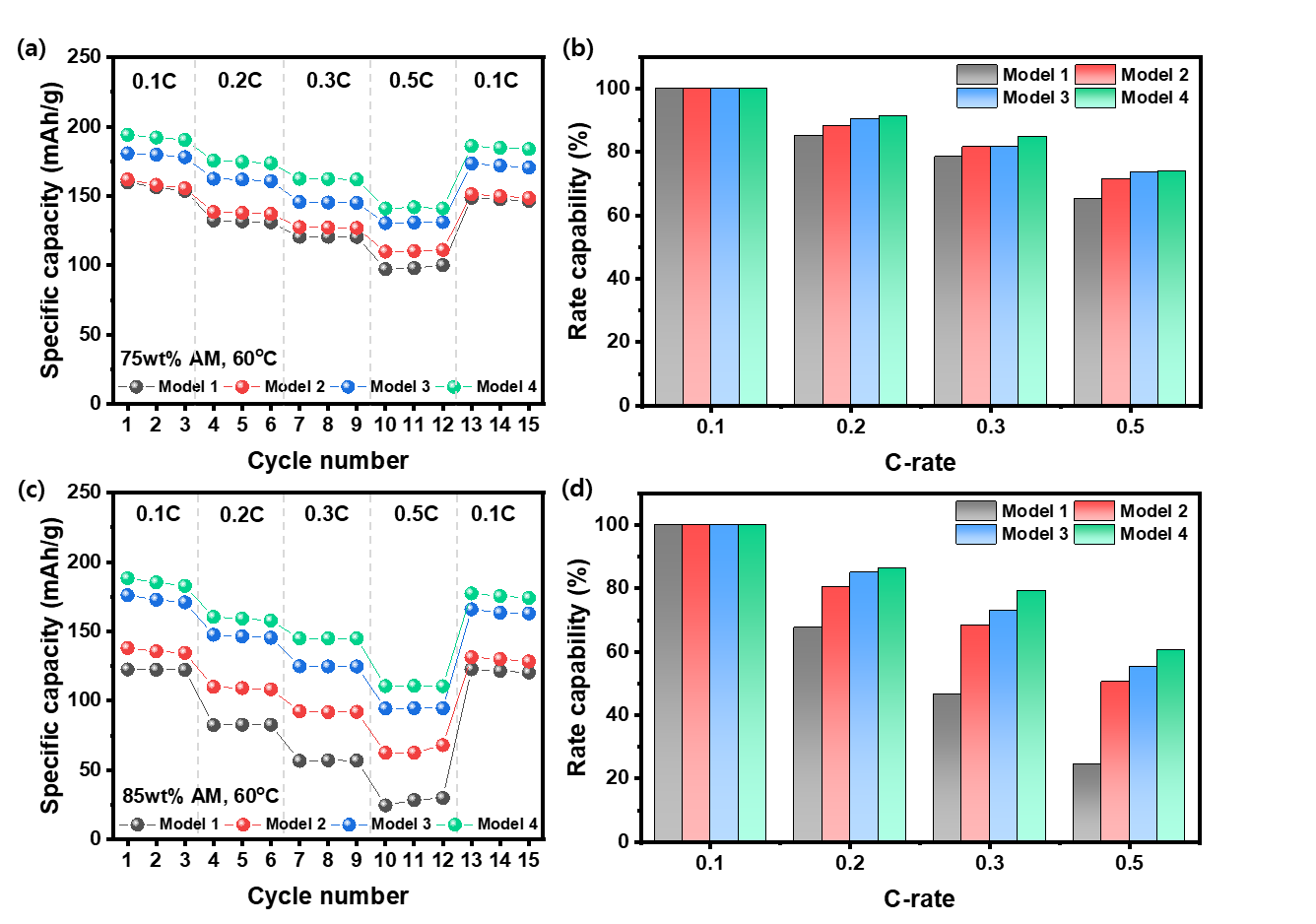


**Fig. S16** ASSB cell performance with 75wt% AM mass loading; **a** rate performance, **b** rate capability. ASSB cell performance with 85wt% AM mass loading; **c** rate performance, **d** rate capability at 60 °C


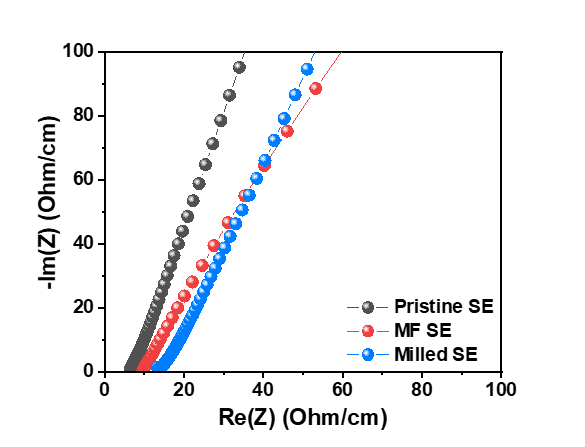


|  | **Pristine SE** | **MF SE** | **Milled SE** |
| --- | --- | --- | --- |
| R_1_ (Ohm)  [Bulk] | 1.115 | 1.523 | 1.573 |
| R_2_ (Ohm)  [Grain Boundary] | 5.505 | 7.675 | 13.4 |
| Total resistance (Ohm) | 6.620 | 9.198 | 16.973 |
| Ionic conductivity (mS cm^-1^) | 14.82 | 10.53 | 5.704 |

**Fig. S17** Nyquist plots pristine SE, SE after mechanofusion process (conditions: 2000rpm, 1h), milled SE (conditions: 500rpm, 3h, 5mm ZrO_2_ ball) at 60 °C


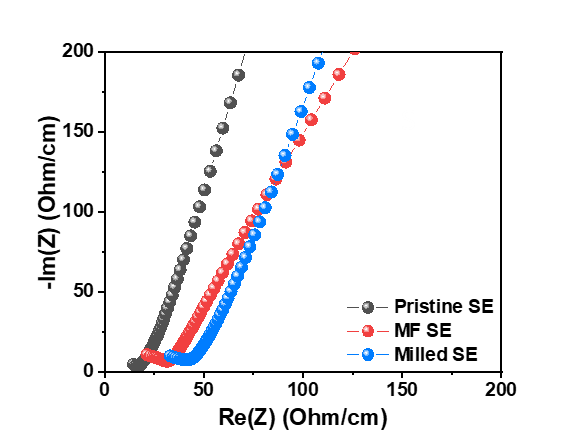


|  | **Pristine SE** | **MF SE** | **Milled SE** |
| --- | --- | --- | --- |
| R_1_ (Ohm)  [Bulk] | 3.38 | 5.00 | 21.64 |
| R_2_ (Ohm)  [Grain Boundary] | 14.55 | 26.85 | 21.9 |
| Total resistance (Ohm) | 17.83 | 31.85 | 43.54 |
| Ionic conductivity (mS cm^-1^) | 5.499 | 3.040 | 2.224 |

**Fig. S18** Nyquist plots pristine SE, SE after mechanofusion process (conditions: 2000rpm, 1h), milled SE (conditions: 500rpm, 3h, 5mm ZrO_2_ ball) at 30$℃$


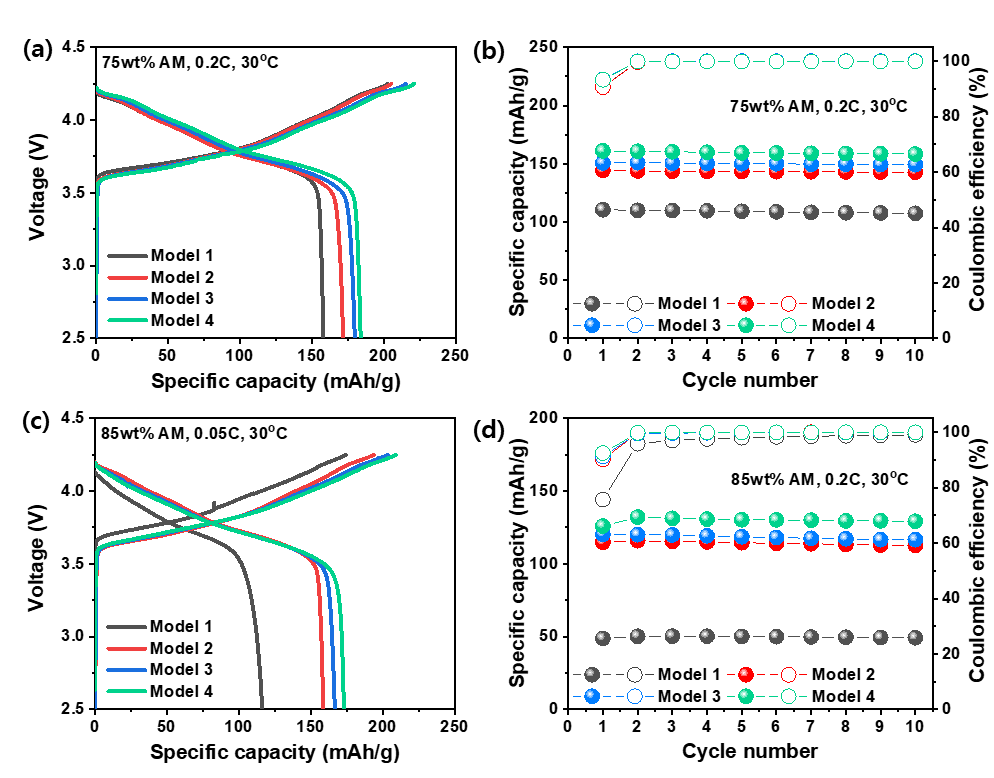


**Fig. S19** Models with 75wt% AM loading: **a** Initial charge and discharge curves at 0.05C (1C=200mA g-1) and **b** Cycle retention at 0.2C (1C=200mA g^-1^) at 30℃ for all-solid-state cathode model/Li metal half-cell; Models with 85wt% AM loading: **c** Initial charge and discharge curves at 0.05C (1C=200mA g^-1^) and **d** Cycle retention at 0.2C (1C=200mA g^-1^) at 30℃ for all-solid-state cathode model/Li metal half-cell


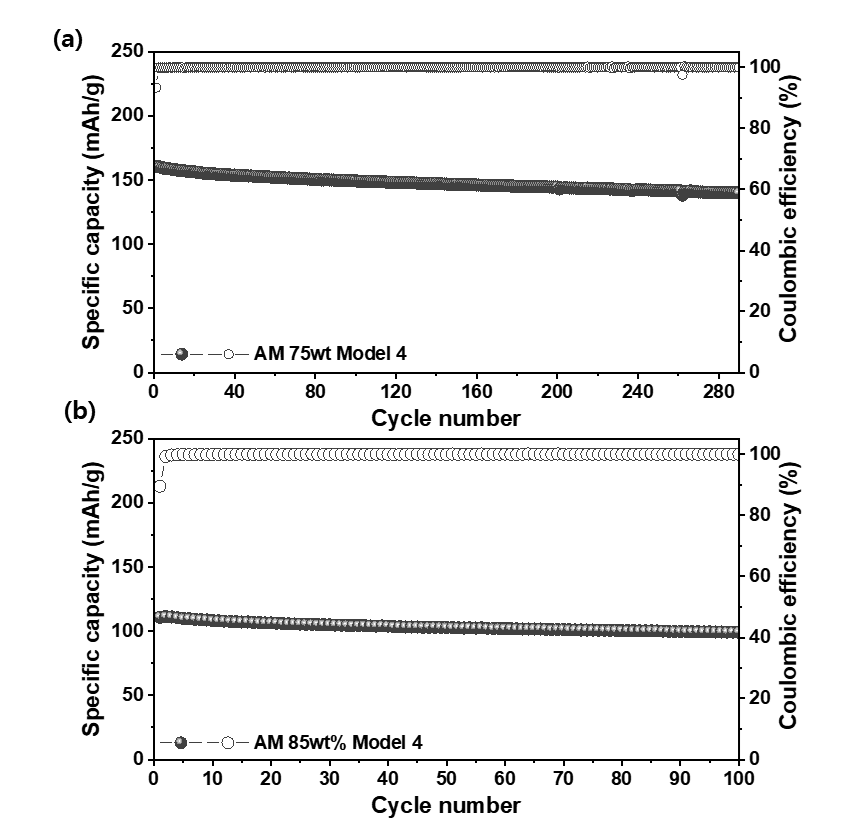


**Fig. S20** Cycle retention at 0.2C(1C=200mA g^-1^) for Model 4 with **a** 75wt% AM loading, **b** 85wt% AM loading


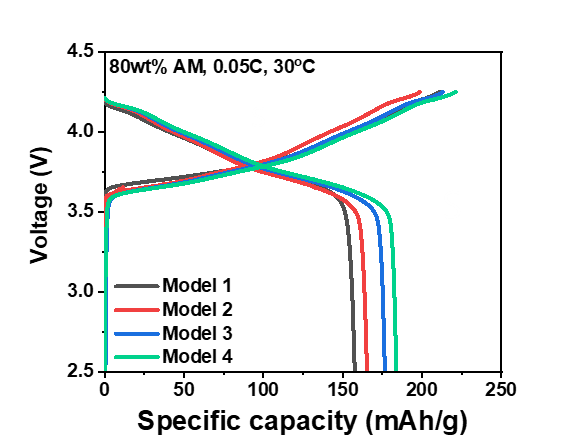


**Fig. S21** Initial charge and discharge curves at 0.05C (1C=200mA g^-1^) for all-solid-state cathode model/Li metal half-cell with 80wt% AM loading at 30$℃$

| **AM content** | **Initial capacity**  **(mAh g^-1^)** | **Initial CE**  **(%)** |
| --- | --- | --- |
| 75wt% | 180 | 81.2 |
| 80wt% | 178 | 81.7 |
| 85wt% | 157 | 74.3 |


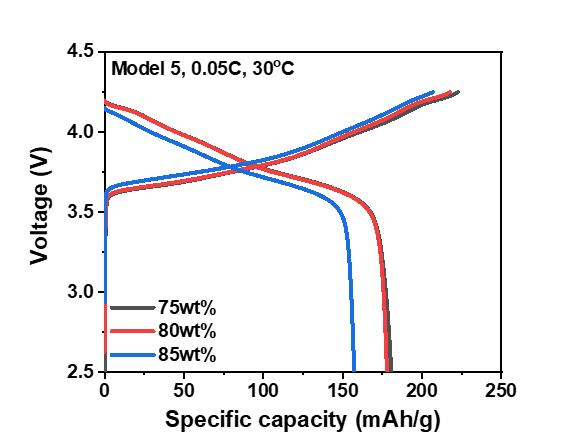


**Fig. S22** Initial charge and discharge curves at 0.05C (1C=200mA g^-1^) for all-solid-state cathode model/Li metal half-cell at 30$℃$ (cathode model mixed by Model 1 mixing protocol with small SE particles)

The volumetric energy density and gravimetric energy density of a battery can be simply calculated by following formula, respectively:

$$Volumetric energy density= \frac{Voltage\left( V \right)\times Capacity\left( mAh \right)}{Volume\left( {cm}^{3} \right)}= \frac{Wh}{L}$$

$$Gravimetric energy density= \frac{Voltage\left( V \right)\times Capacity(mAh)}{Weight(kg)}= \frac{Wh}{kg}$$

The voltage is a nominal voltage of NCM811 and volume is corresponding to total volume of cathode, separator and counter electrode (Li metal in this study). The capacity is determined from the following formula:

$Capacity\left( mAh \right)=Specific capacity\left( \frac{mAh}{g} \right) \times AM loading mass(g)$

**Table S5** Volumetric energy density (Wh L^-1^) and gravimetric energy density (Wh kg^-1^) comparison of Models 1~4

| **Model** | **AM loading** | **Volumetric density**  **(Wh L^-1^)** | **Gravimetric density**  **(Wh kg^-1^)** | **Volumetric density**  **(Wh L^-1^)** | **Gravimetric density**  **(Wh kg^-1^)** |
| --- | --- | --- | --- | --- | --- |
|  |  | **30**$\boldsymbol{℃}$ | | **60**$\boldsymbol{℃}$ | |
| Model 1 | 75 wt% | 765 | 251 | 863 | 280 |
|  | 85 wt% | 640 | 209 | 735 | 220 |
| Model 2 | 75 wt% | 853 | 272 | 903 | 287 |
|  | 85 wt% | 893 | 285 | 1016 | 297 |
| Model 3 | 75 wt% | 913 | 285 | 1000 | 312 |
|  | 85 wt% | 1042 | 299 | 1123 | 324 |
| **Model 4** | **75 wt%** | **940** | **291** | **1042** | **324** |
|  | **85 wt%** | **1094** | **311** | **1258** | **357** |

***** The anode thickness of 30$\mu m$ and separation layer thickness of 30$\mu m$ were assumed. ***** The anode weight of 13.6 mg and separation layer weight of 4.4 mg were assumed.

**Table S6** Volumetric energy density (Wh L^-1^) comparison of other reported in ASSBs with this work

| **Cathode** | **AM content** | **Loading level (thickness)** | **Capacity**  **(C-rate)** | **Temperature** | **Volumetric energy density*** | **Refs.** |
| --- | --- | --- | --- | --- | --- | --- |
| NCM622 | 79.2 wt% | 28 mg cm^-2^  (117$\mu m$) | 146 mAh g^-1^  (0.1C) | 30$℃$ | 676 Wh L^-1^ | 26 |
| NCM811 | 80 wt% | - | 142 mAh g^-1^ (0.1C) | 30$℃$ | 516 Wh L^-1^ | 27 |
| NCA | 70 wt% | 11.3 mg cm-^2^  (50$\mu m$) | 202 mAh g^-1^ (0.1C) | 30$℃$ | 860 Wh L^-1^ | 55 |
| LCO | 70 wt% | 19.9 mg cm^-2^  (90$\mu m$) | 115 mAh g^-1^ (0.1C) | 25$℃$ | 607 Wh L^-1^ | 41 |
| NCM622 | 70 wt% | 1.9 mAh cm^-2^  (90$\mu m$) | 153 mAh g^-1^ (0.1C) | 30$℃$ | 561 Wh L^-1^ | 43 |
| NCM333 | 76.2 wt% | -  (71$\mu m$) | 151 mAh g^-1^ (-) | 30$℃$ | 993 Wh L^-1^ | 56 |
| NCM333 | 66 wt% | 9.5 mg cm^-2^  (84$\mu m$) | 158 mAh g^-1^ (C/24) | 25$℃$ | 819 Wh L^-1^ | 57 |
| NCM622 | 70 wt% | 15.3 mg cm^-2^  (90$\mu m$) | 148 mAh g^-1^ (C/10) | 25$℃$ | 737 Wh L^-1^ | 58 |
| NCM622 | 70 wt% | 7 mg cm^-2^  (50$\mu m$) | 170 mAh g^-1^ (0.1C) | 55$℃$ | 724 Wh L^-1^ | 59 |
| NCM622 | 67.2 wt% | 11 mg cm^-2^  (26$\mu m$) | 140 mAh g^-1^ (C/50) | 60$℃$ | 424 Wh L^-1^ | 60 |
| NCM85 | 84.8wt% | -  (200$\mu m$) | 203 mAh g^-1^  (0.01C^2^) | 25$℃$ | 786 Wh L^-1^ | 61 |
| **NCM811** | **85 wt%** | **30.6 mg cm^-2^**  **(93**$\boldsymbol{\mu m}$**)** | **173 mAh g^-1^ (0.05C)** | **30**$\mathbf{℃}$ | **1094** **Wh L^-1^** | **This work** |
|  |  |  | **199 mAh g^-1^ (0.05C)** | **60**$\mathbf{℃}$ | **1258** **Wh L^-1^** |  |
| ***** The anode thickness of 30$\mu m$ and separation layer thickness of 30$\mu m$ were assumed**.** | | | | | | |

**Supplementary References**

[S1] U.H. Kim, T.Y. Yu, J.W. Lee, H.U. Lee, L. Belharouak et al., Microstructure- and interface-modified Ni-rich cathode for high-energy-density all-solid-state lithium batteries. ACS Energy Lett. **8**(1), 809-817 (2023).

<https://doi-org-ssl.oca.korea.ac.kr/10.1021/acsenergylett.2c02715>
